# Two Birds, One Stone: The Effectiveness of Health and Environmental Messages to Reduce Meat Consumption and Encourage Pro-environmental Behavioral Spillover

**DOI:** 10.3389/fpsyg.2020.577111

**Published:** 2020-10-07

**Authors:** Emily Wolstenholme, Wouter Poortinga, Lorraine Whitmarsh

**Affiliations:** ^1^School of Psychology, Cardiff University, Cardiff, United Kingdom; ^2^Welsh School of Architecture, Cardiff University, Cardiff, United Kingdom; ^3^Department of Psychology, University of Bath, Bath, United Kingdom

**Keywords:** meat, health, environment, spillover, message, intervention, identity

## Abstract

There is a growing consensus that reducing excess meat consumption will be necessary to meet climate change targets, whilst also benefitting people’s health. Strategies aimed at encouraging reduced meat consumption also have the potential to promote additional pro-environmental behaviors through behavioral spillover, which can be catalyzed through an increased pro-environmental identity. Based on this, the current study tested the effectiveness of a randomized two-week messaging intervention on reducing red and processed meat consumption and encouraging pro-environmental behavioral spillover. Participants were undergraduate students in the United Kingdom (*n* = 320 at baseline) randomly allocated to four conditions in which they received information about the health, environmental, or combined (health and environmental) impacts of meat consumption, and a no-message control. The results showed that receiving information on the health and/or environmental impacts of meat was effective in reducing red and processed meat consumption compared to the control group during the intervention period, with some effects remaining one-month later. However, the intervention did not have any effect on pro-environmental identity and there was little evidence of behavioral spillover. Implications for future research and interventions aimed at reducing meat consumption are discussed.

## Introduction

Most people in high-income countries eat high amounts of meat that exceed nutritional needs (Sans and Combris, 2015), while meat consumption in lower income countries is also on an upward trajectory (Tilman and Clark, 2014). Though differences are found according to country and commodity, recent data shows that the consumption of meat remains high in many countries (see OECD, 2020). This is problematic given that the overconsumption of meat is associated with serious negative health and environmental impacts. For example, the overconsumption of red and processed meat is associated with an increased risk of non-communicable diseases; cardiovascular disease, stroke and certain forms of cancer (Walker et al., 2005; Micha et al., 2012; Yang et al., 2016). Furthermore, meat is a major driver of climate change, responsible for approximately 15% of global anthropogenic greenhouse gas emissions (Gerber et al., 2013; Bailey et al., 2014). This has led to a growing consensus that reducing excess meat consumption will be necessary to meet climate change targets, whilst also benefitting people’s health (e.g., Bajželj et al., 2014; Hedenus et al., 2014; Tilman and Clark, 2014; Ritchie et al., 2018). However, attempts to reduce meat consumption remain absent from most climate change mitigation strategies, given that such strategies have low political appeal and may be unpopular among the public (e.g., Laestadius et al., 2014). This has subsequently led to a lack of media attention and low public awareness of the link between meat consumption and climate change in many countries (Wellesley et al., 2015). Indeed, people tend to greatly underestimate the extent to which meat production contributes to climate change (Bailey et al., 2014; Macdiarmid et al., 2016). This is concerning, given that people’s willingness to reduce their meat consumption has been associated with the extent to which they believe reducing their meat consumption will be effective in mitigating climate change (Truelove and Parks, 2012; de Boer et al., 2016). The lack of awareness of the environmental impacts of meat eating therefore may be contributing to people’s inaction (Bailey et al., 2014; Wellesley et al., 2015). There is therefore a clear need to communicate the negative impacts of meat, including its contribution to climate change, to raise awareness and motivate individuals to reduce their consumption.

Intervention studies aimed at reducing meat consumption have begun to emerge in the literature in recent years. These studies have demonstrated that information provision can be effective in encouraging individuals to reduce their meat consumption (e.g., see Bianchi et al., 2018). Although, it should be noted that much of the literature has focused on the effectiveness of interventions on changing attitudes or intentions to eat meat, while fewer studies have demonstrated the effect of informational strategies on eliciting behavior change, i.e., reducing meat consumption (see Harguess et al., 2019). This is problematic as attitudes and intentions do not always predict behavior (Kormos and Gifford, 2014; Hassan et al., 2016), including reducing ones’ meat consumption (Allen and Baines, 2002; Stubbs et al., 2018). Much of the literature has focused on the effectiveness of health messages (e.g., Berndsen and Van Der Pligt, 2005; Cordts et al., 2014; Bertolotti et al., 2019), while fewer studies have demonstrated the effectiveness of environmental messages (e.g., Hunter and Röös, 2016; Graham and Abrahamse, 2017; Stea and Pickering, 2019), in encouraging meat reduction. This possibly stems from evidence that individuals tend to underestimate the impact of meat on contributing to climate change and tend to be unwilling to reduce their meat consumption for environmental reasons (De Boer et al., 2013; Macdiarmid et al., 2016). This has led some authors to conclude that focusing on the health impacts might be a more effective strategy for encouraging a reduced meat consumption (e.g., Wellesley et al., 2015). However, studies comparing the effects of health and environmental messages are limited, and while there is some evidence that health messages can be more effective in increasing intentions to reduce one’s meat consumption (e.g., Cordts et al., 2014), other studies have found no significant differences in the effectiveness of health and environmental messages on reducing meat consumption (e.g., Carfora et al., 2019b).

On the other hand, it is possible that multiple arguments can be combined to encourage a reduced meat consumption. This is based on evidence that pro-environmental behavior, including decisions to reduce one’s meat consumption, can be motivated by many different factors (Jagers et al., 2017). Thus, it has been suggested that combining different motives might be a more effective strategy for reducing meat consumption than communicating each of these issues in isolation (De Boer et al., 2013). As such, recent literature has begun to investigate whether providing information on different impacts of meat simultaneously, can be an effective strategy for reducing consumption. For example, Amiot et al. (2018) tested the effect of a multi-component intervention, part of which included providing information highlighting the impacts of meat on health, the environment and on animal welfare, on reducing meat consumption among Canadian male participants. They found no differences two weeks after receiving the information, however, four weeks later, participants in the experimental condition ate significantly less red meat than those in the control group. On the other hand, Carfora et al. (2019b) recently tested the effects of providing information on the health and environmental impacts of meat both separately and in combination, as part of a randomized messaging intervention in Italy. They found that providing information either about the health or the environmental impacts of meat was effective in reducing participants’ red and processed meat consumption shortly after the intervention and one-month later, while combining this information had no significant effects. Thus, it is not clear whether combining different types of information would be an effective strategy for encouraging a reduced meat consumption compared to communicating this information in isolation.

Interventions aimed at encouraging reduced meat consumption could also have the potential to encourage other untargeted pro-environmental behaviors, through pro-environmental behavioral spillover. Positive behavioral spillover occurs when adopting an initial pro-environmental behavior leads to a greater engagement in other subsequent pro-environmental behaviors (Poortinga et al., 2013). Spillover is most likely to occur between similar behaviors (Whitmarsh and O’Neill, 2010) and can occur between both private-sphere (i.e., consumer or domestic) and public-sphere (i.e., political or social) behaviors (Nash et al., 2017). For example, Lanzini and Thøgersen (2014) found that an intervention aimed at encouraging green purchasing behavior also led to an increase in recycling, public transport use, as well as water and energy saving behaviors. Furthermore, Thomas et al. (2019) found that a charge on plastic bags lead to an increased use of re-usable shopping bags and increased support for other waste-related policies. Thus, an intervention aimed at one behavior has the potential to catalyze other lifestyle changes, maximizing the positive outcomes of an intervention on the environment. However, negative spillover can also occur, whereby successfully encouraging an individual to adopt a pro-environmental behavior is associated with a decreased willingness to perform other pro-environmental behaviors, or an increase in environmentally unsustainable behaviors due to contribution ethic or moral licensing effects (Thøgersen and Crompton, 2009). For example, Tiefenbeck et al. (2013) found that households who reduced their water consumption following an intervention aimed at water conservation subsequently increased their energy consumption, compared to a control group. Thus, negative spillover has the potential to undermine efforts to promote environmentally friendly action. Little is currently known about whether an intervention aimed at meat reduction would lead to behavioral spillover, or whether any potential spillover effects would be positive or negative. Considering that few studies have investigated the effects of interventions on reducing people’s meat consumption, even fewer have investigated whether a reduced consumption of meat might be associated with uptake of other pro-environmental behaviors.

Despite this, two recent studies show some evidence of positive behavioral spillover following an intervention aimed at meat reduction. Verfuerth et al. (2019) investigated spillover following a workplace intervention which used information provision on the environmental impacts of meat to encourage a reduced meat consumption. They found that individuals who reduced their meat consumption during the intervention were also more likely to engage in other pro-environmental behaviors outside the workplace, including buying local rather than imported food produce, recycling, eating smaller food portions, reducing packaging, and buying products with sustainable palm oil one-month later. Another study found that participants who reduced their red meat consumption as part of a message-framing intervention showed an increased environmental concern, which in turn lead to an increased likelihood of donating to an environmental organization (Carrico et al., 2018). This effect was found for participants who had received information on the health impacts of meat, as well as those who had received information on the environmental impacts. Thus, while the literature investigating meat reduction and behavioral spillover is in its infancy, there is some evidence that an intervention aimed at meat reduction could potentially lead to an uptake of other private- and public-sphere pro-environmental behaviors. Furthermore, the literature suggests that this effect can occur even if meat reduction is motivated by health rather than environmental motives (e.g., Carrico et al., 2018).

However, there is evidence that different types of information can either promote or dampen pro-environmental behavior and subsequent spillover effects. For example, Schwartz et al. (2015) found that participants were more willing to enroll in an energy-saving program when the environmental benefits were emphasized compared to financial benefits, and also compared to when both the financial and environmental benefits were emphasized together. Furthermore, participants were less likely to cite environmental reasons for enrolling in an energy saving program when it was framed in terms of the financial benefits, even when these benefits were emphasized together with the environmental benefits. Similarly, Evans et al. (2012) found that participants were more likely to recycle a sheet of paper following a task highlighting the environmental aspects of a behavior (car-sharing) compared to a control condition. However, there was no effect when financial aspects of the behavior were highlighted, even if the financial aspects were highlighted together with the environmental aspects in a combined condition. The authors concluded that highlighting the environmental impacts of a behavior would make self-transcendent values (e.g., helping others and the environment) more salient leading to further related actions, while highlighting the financial impacts of the behavior would have made self-interest values (e.g., power and wealth) salient, increasing the likelihood of other self-interest rather than self-transcendent behaviors. This is in line with goal-framing theory which indicates that spillover results from the activation of a common motivation or overarching goal, e.g., to mitigate rising greenhouse gas emissions, which can cause an indirect link between different behaviors (Lindenberg and Steg, 2007). On the other hand, the evidence suggests that highlighting other goals, particularly relating to self-interest, might reduce the likelihood of spillover occurring. While there is evidence that health-framed messages may have more universal appeal than environmentally framed ones (Myers et al., 2012), very little research has explored the effects of combining health and environmental messages (Carfora et al., 2019b). Thus, it is not clear whether combining health with environmental messages might have a positive or negative effect on spillover.

There is widespread evidence to suggest that pro-environmental self-identity plays an important role in pro-environmental behavior. For example, pro-environmental identity has been found to predict pro-environmental behavior over and above other psychosocial factors (Whitmarsh and O’Neill, 2010). People can make inferences about their identity based on past behavior, which may subsequently lead people to act in accordance with that self-perception (Bem, 1972). For example, reminding individuals of their past pro-environmental behavior can lead to an increased pro-environmental identity and as a result a greater engagement in subsequent pro-environmental behaviors (see Cornelissen et al., 2008; Van der Werff et al., 2013). Self-identity is therefore considered a key factor in behavioral spillover (Whitmarsh and O’Neill, 2010; Truelove et al., 2014). In line with this, Verfuerth et al. (2019) found that participants who reduced their meat consumption during a workplace intervention showed an increased pro-environmental identity, which was associated with positive spillover to pro-environmental behavior outside of the workplace. Thus, it is important to consider pro-environmental identity when investigating behavioral spillover, as it can act as an important catalyst for pro-environmental behavior.

Finally, studies have shown that combining information with other techniques can be effective for increasing the efficacy of interventions aimed at reducing meat consumption. For example, framing information in terms of social values (e.g., self-transcendence or self-enhancement) can increase positive attitudes toward eating less meat when matched to the existing values of participants (Graham and Abrahamse, 2017). Pairing information with implementation intentions, for example a clear time-oriented goal as to how and when one will change their behavior, can improve the efficacy of interventions aimed at reducing meat consumption (e.g., Amiot et al., 2018; Rees et al., 2018). Additionally, encouraging participants to self-monitor their meat intake can also be used increase the likelihood that a goal to reduce ones’ consumption is achieved (e.g., Carfora et al., 2017, 2019b). Food diaries are also often used to encourage a greater adherence with dietary programs and to increase awareness of ones’ food choices (Zepeda and Deal, 2008). As such, food diaries can be used to encourage a reduced meat consumption when combined with other techniques, such as self-monitoring (e.g., Carfora et al., 2017, 2019b; Amiot et al., 2018). Thus, the literature suggests that providing information on the different impacts of meat can be effective in encouraging meat reduction, especially when combined with other intervention components.

The current study builds on existing literature, to further investigate the effects of information provision on reducing red and processed meat consumption and encouraging pro-environmental behavioral spillover. Whereas past literature has tended to focus on the effects of interventions on attitudes and intentions, we test the effects of information (coupled with goal intentions) on red and processed meat consumption reported across three time points. We build on past literature by investigating the effects of environmental, health and combined messages to reduce red and processed meat consumption. Furthermore, we add to the emerging literature on behavioral spillover, by investigating whether eating less red and processed meat would spillover to other untargeted pro-environmental behaviors, whether any spillover effects could be attributed to an increased pro-environmental identity, and whether spillover and pro-environmental identity might differ across the different messaging conditions. The potential for spillover is also examined across various public- and private-sphere pro-environmental behaviors, to shed light on the types of pro-environmental behaviors that might occur following a reduced red and processed meat consumption. Overall, this study aims to improve understanding of the potential effectiveness of informational strategies on encouraging dietary change and eliciting other pro-environmental lifestyle choices.

Based on the literature reviewed above, we hypothesized that participants receiving information on the health or environmental impacts of meat would significantly reduce their red and processed meat consumption during the intervention and one-month later, as compared to baseline and control participants (H1). It was not known whether participants who received combined information on both the health and environmental impacts would reduce their red and processed meat consumption at either time point, given that previous studies have yielded mixed results on the effects of combined messages (Research Question 1 – RQ1). Second, it was hypothesized that reduced consumption of red and processed meat would lead to an increased willingness to perform other pro-environmental behaviors immediately after the intervention and one-month later (H2). Third, it was hypothesized that this hypothesized relationship would be mediated by pro-environmental identity, whereby reduced consumption of red and processed meat would lead to an increased pro-environmental identity (H3), in turn increasing willingness to perform other pro-environmental behaviors when controlling for change in red and processed meat consumption (H4). We also explored whether the different messaging conditions would have an effect on pro-environmental identity and behavioral spillover. Specifically, we explored whether participants in the environment, health and combined conditions would be more willing to perform untargeted pro-environmental behaviors compared to participants in the control condition (Research Question 2 – RQ2) and whether these participants would also show a greater change in their pro-environmental identity compared to participants in the control condition (Research Question 3 – RQ3).

## Materials and Methods

### Ethics

This study was reviewed and approved by the Cardiff University School of Psychology Research Ethics Committee.

### Participants

Participants were recruited from a university in the United Kingdom. The study was advertised on posters placed in university buildings, as well as through online social media pages and an online participant pool for Psychology undergraduate students. In all cases, the study was advertised as being “a Psychology project about attitudes and food choice.” In the information sheet, the study was described as being about “attitudes and red meat” specifically. Participants were not informed that the study aimed to investigate an intervention for reducing red and processed meat consumption. Eligibility criteria were included so that only students who consumed at least three portions of red or processed meat each week and were not already following any specific dietary plan qualified for participation. Where the study was advertised, it was stated that only students who consumed at least three portions of red or processed meat would be eligible to take part. Participants were also required to confirm that they met each of the inclusion criteria via screening questions at the start of the survey. Those that did not meet all criteria were automatically directed to the end of the survey and were disqualified from participation. A power analysis using G^∗^power (for mac version 3.1.9.4) was conducted to determine the required sample size to detect changes in meat consumption between the different conditions over time. The analysis was based on a small-medium effect size (η^2^ = 0.30), determined by similar past literature investigating the effectiveness of interventions aimed at reducing meat consumption (Carfora et al., 2017; Amiot et al., 2018). With a power of 0.95 and α = 0.05, the results showed that a sample size of 250 participants was needed. We used this as a guideline and oversampled in anticipation of participant drop-outs. In total, 320 participants took part at baseline in exchange for payment (£15) or course credits; 59 participants were male and 260 female, and one participant for which gender information was missing. At baseline, the sample involved 293 undergraduate and 27 postgraduate students, with a mean age of 20 years. At time 2 (end of the intervention), 251 (78%) participants answered the survey, of which 205 were female and 45 were male, the gender information was missing for one participant. At this time, 229 participants were undergraduate and 22 were postgraduate students, the mean age of participants was 20. At time 3 (one month after the intervention), 238 (74%) participants answered the survey, of which 191 were female and 46 were male, the gender information missing for one participant. At this time, 217 participants were undergraduates and 21 participants were postgraduate students, the mean age of participants was 20.

### Design

The study used a mixed design. A between-subjects design randomly allocated participants to one of four conditions: (1) Health (T1: *n* = 78; T2: *n* = 58, T3: *n* = 56), in which participants received information on the impacts of red and processed meat on health. (2) Environment (T1: *n* = 83; T2: *n* = 67; T3: *n* = 67), in which participants received information on the impacts of red and processed meat on the environment. (3) Combined (T1: *n* = 86; T2: *n* = 69; T3: *n* = 63), in which participants received information on the impacts of red and processed meat on both health and on the environment. (4) Control (T1: *n* = 73; T2: *n* = 57; T3: *n* = 52), in which participants did not receive any information on the impacts of meat. Participants in the health, environment and combined conditions were also provided with a time-oriented goal, to try to eat no more than two portions of red/processed meat each week for the two-weeks of the intervention period. Participants in the control condition were asked not to change their diet in anyway. The information displayed to participants can be viewed in the Supplementary Appendix S1. Red and processed meat consumption was compared over time using a within-subjects design, as well as between conditions using a between-subjects design.

A within-subjects design was used to investigate the relationship between participants’ willingness to perform additional pro-environmental behaviors as a result of red and processed meat reduction and an increased pro-environmental identity. A between-subjects design was used to compare participants’ willingness to preform additional pro-environmental behaviors and change in pro-environmental identity between conditions.

### Materials

#### Online Survey(s)

The study was conducted online through a series of surveys implemented on Qualtrics and an automated chatbot using Facebook messenger. The pre-test survey was given to participants at baseline (T1) before the messaging intervention. This survey included a consent form and information sheet, demographic questions, and a measure of red and processed meat consumption. The survey also included a link to the automated Facebook chatbot, from which the randomized messaging intervention was implemented. The post-test survey was sent to participants at the end of the two-week messaging intervention (T2) and included a measure of red and processed meat consumption, a measure of behavioral spillover and a measure of pro-environmental identity. The same survey was sent to participants again at the one-month follow-up (T3).

#### Food Diaries

Participants were asked to record all their food intake using a food diary every day during the two-week intervention period, to increase engagement with the intervention programme. The food diaries were implemented via a survey on Qualtrics which was sent through a link in the Facebook chatbot each day of the two-week intervention period. Participants were asked to indicate which foods they had eaten throughout the day for breakfast, lunch, dinner, as well as any snacks. Participants could select which foods they had eaten from a list of response items (e.g., cereals, beans, red meat etc.) and had the option to enter free text for any foods not included within the provided response items. For each food, participants were required to indicate the number of portions consumed, as well as the portion size from “small,” “medium,” and “large.” The food diaries were used during the two-week intervention period but were not used at baseline or after the intervention. The data from the food diaries are not used in the current paper, as comparisons cannot be made from before to after the randomized messaging intervention.

#### Randomized Messaging Intervention

The intervention was run through an automated private chat on Facebook Messenger, which was built using “ManyChat” chatbot software (Manychat.com). Every day for two weeks, participants in the health, environment and combined conditions received messages on the positive impacts of eating less red and processed meat on health, the environment, or on both health and the environment. This was followed by a reminder to try not to eat more than two portions of red and processed meat each week, in addition to a reminder to complete the food diary. For example, in the environment condition, one message read: “*If you eat only a small amount of red and processed meat, you will protect the environment by reducing excessive land use. Remember to try and eat no more than two portions of red and processed meat this week. Please record all of the food you have eaten today using today’s food diary*.” The messages highlighted a different health and/or environmental issue each day of the intervention. Thus, participants in the health, environment and combined conditions received 14 different messages in total. The messages were sent to participants once in the morning (at 8 am) and once in the evening (at 5 pm), every day during the two-week intervention period. Control participants were not sent any information on the impacts of meat but were sent a reminder to complete the food diary every day of the intervention e.g., “*Please record all of the food you have eaten today using today’s food diary*,” once in the morning (8 am) and again in the evening (5 pm) every day during the intervention period. The messages sent to participants each day of the intervention can be viewed in the Supplementary Appendix S2.

### Measures

#### Red and Processed Meat Consumption

Self-reported red and processed meat consumption was recorded using a measure adapted from existing literature (Carfora et al., 2019a,b). Red and processed meat consumption was measured separately. For each type of meat, participants were provided with a definition (e.g., “*Processed meat includes meat that has been modified to improve its taste or shelf life through smoking, curing or adding salt or preservatives*…”) and were given an example of a medium portion size in grams (e.g., “*A medium portion refers to about 60 grams, for example two small sausages or five slices of salami*…”). Red and processed meat consumption was recorded at three time points: at baseline, immediately after the two-week intervention period, and one-month later. At each time point, participants were asked to record the number of servings of red and processed meat they had consumed during the *previous week* (e.g., “*How many servings of processed meat have you eaten in the previous week? If you cannot remember please give your best estimate*”), using a 15-point response scale from 0 to 14 servings or more. Thus, the measures reflect the number of servings consumed by participants during one week before the intervention (T1), during the second week of the intervention period (T2) and four weeks after the intervention (T3). Responses for red and processed meat consumption were combined to create a single outcome variable.

#### Behavioral Spillover

Participants’ willingness to perform ten different pro-environmental behaviors was measured at T2 and T3, as an indicator of behavioral spillover. Participants were asked how often they planned to perform the following behaviors in the following 6 months: *“have shorter showers or infrequent baths*,*” “Purchase an eco-friendly product*,*” “buy a product with less packaging*,*” “buy organic food produce*,*” “Buy local rather than imported food produce*,*” “eat seasonal fruit and vegetables*,*” “reduce my consumption of meat and dairy products*,*” “use public transport instead of driving my car*,*” “volunteer for an environmental group*,*”* and *“donate to an environmental group.”* For each item, participants were asked to select one of the following options: “not at all,” “once,” “2 to 3 times,” “4 to 5 times,” “6 to 7 times,” “8 to 9 times” or “more than 10 times.” Responses were coded from 1 (“not at all”) to 7 (“more than 10 times”). This measure was adapted from previous literature on behavioral spillover (e.g., Whitmarsh and O’Neill, 2010; Lauren et al., 2017).

#### Pro-environmental Identity

Pro-environmental identity was measured at T1, T2, and T3 using a three-item scale adapted from Whitmarsh and O’Neill (2010): “*I am an environmentally-friendly person*,” “*I am someone who is concerned with environmental issues*” and “*I would be embarrassed to be seen as having an environmentally-friendly lifestyle*” (reverse coded). The third item was removed after reliability analysis indicated doing so would significantly improve the reliability of this measure (from *a* = 0.63 to *a* = 0.84 at T1, from *a* = 0.67 to *a* = 0.80 at T2 and from *a* = 0.63 to *a* = 0.80 at T3). Items were presented as 7-point Likert scales ranging from 1 = strongly disagree to 7 = strongly agree. Pro-environmental identity was also measured through a visual scale adapted from the Inclusion of Nature in Self scale (Schultz, 2001). Participants were given a brief description of “*an environmentally conscious person*” and were asked to select one of seven images, each depicting a pair of circles representing (1) the self and (2) an “environmentally conscious person,” with varying degrees of overlap. Responses were coded from 1 (no overlap between the circles) to 7 (complete overlap of the circles). Both scale and visual measures were combined to capture different aspects of identity and considering that using multiple heterogenous items within a scale can increase its validity (e.g., Eisinga et al., 2013). The overall measure of pro-environmental identity showed good reliability at T1 (*a* = 0.82), T2 (*a* = 0.80) and at T3 (*a* = 0.80).

### Procedure

The study was conducted entirely online using Qualtrics and Facebook Messenger. Participants were sent a link to complete the baseline survey and were directed to answer screening questions, followed by demographic questions and a measure of red and processed meat consumption for the preceding week. Following this, participants were randomly allocated to one of the four messaging conditions using a randomized display logic in Qualtrics. Participants were given a link to the automated chatbot on Facebook Messenger and were told that for the next study phase they would be required to complete a food diary every day for two-weeks. Control participants were asked not to change their diet during this time. Participants in the experimental conditions were given some brief information highlighting the negative impacts of red and processed meat on either health and/or the environment (depending on the condition) and were asked to try to eat no more than two portions of red and processed meat each week of the two-week intervention period. All participants were asked to answer the surveys and food diaries honestly and were told that there were no “right or wrong” answers. The baseline survey ended after participants confirmed they had read and understood this information. The intervention began within one week of completing this survey. Participants were sent automated messages every day during the intervention. On the final day of the two-week intervention period, participants were sent the post-test survey via the Facebook chat and through email. The one-month follow-up survey was sent to participants via the chatbot and email one-month later. Participants were debriefed and then either awarded their credits or paid in cash.

### Statistical Analysis

All data were analyzed using IBM SPSS for mac (version 20). Change in red and processed meat consumption over time was analyzed using a Linear Mixed Model (LMM), which has many advantages over traditional statistical techniques, such as repeated measures ANOVA, including being able to handle missing data without loss of statistical power (Gueorguieva and Krystal, 2004). A hierarchical structure was used with measurement occasion at level one being nested within individuals at level two. Time of the measurement occasion (i.e., whether it is T1, T2, and T3) and condition were included as fixed variables with a time × condition interaction term. Both variables were dummy coded so that the intervention (T2) and at the one-month follow-up (T3) were compared to baseline (T1) as the reference group, and the environment, health and combined conditions were compared to the control condition as the reference group. The effects for time and condition were estimated by constructing fixed slopes and random intercept models with red and processed meat as the dependent variable. This means that the data was modeled assuming that the amount of red and processed meat consumption can vary across the level two units (i.e., individuals), but that the impacts of time and condition are fixed across individuals. Here, we only report the fixed effects for condition and time.

Behavioral spillover was investigated at the end of the intervention period (T2) and one-month later (T3). Participants’ willingness to engage in each of the 10 pro-environmental behaviors was analyzed separately, to shed light on the types of pro-environmental behaviors that might result following reduced red and processed meat consumption. Holm-Bonferroni correction of the *P* value was applied considering the increased risk of type 1 error due to multiple testing of the 10 pro-environmental behaviors at T2 and T3 (Holm, 1979).

## Results

### Pre-analysis

Demographic variables as well as red and processed meat consumption and pro-environmental identity across the different conditions are summarized in Table 1. Analysis was conducted to investigate whether the final participant sample was representative of the initial sample, given the attrition rate (*n* = −82). Chi-square analysis using a Fisher-Freeman-Halton test indicated no significant association between condition and whether participants dropped out of the study (*x*^2^ = 1.45, *p* = 0.698), indicating that the final participant sample was not skewed across conditions. A one-way ANOVA showed that there was no significant difference in participants’ pro-environmental identity [*F*(1,318) = 0.57, *p* = 0.451] or between the amount of red and processed meat consumed by participants [*F*(1,318) = 0.23, *p* = 0.630], in the initial and final samples. Therefore, the results suggest that the final sample of participants was equivalent to the initial sample for the variables of interest.

**TABLE 1:** Participant demographics and variables of interest by condition at T1.

	**Control**	**Health**	**Environment**	**Combined**
Age	M = 20, SD = 2.09	M = 20, SD = 3.30	M = 20, SD = 1.92	M = 20, SD = 1.77
**Gender**				
Male	*N* = 18	*N* = 16	*N* = 15	*N* = 10
Female	*N* = 55	*N* = 62	*N* = 68	*N* = 75
**Level of study**				
Undergraduate	*N* = 64	*N* = 73	*N* = 76	*N* = 80
Postgraduate	*N* = 9	*N* = 5	*N* = 7	*N* = 6
Red and processed meat consumption	M = 7.03, SD = 3.23	M = 7.59, SD = 3.57	M = 7.01, SD = 3.25	M = 7.35, SD = 3.66
Pro-environmental identity	M = 4.71, SD = 1.05	M = 4.52, SD = 1.17	M = 4.43, SD = 0.867	M = 4.51, SD = 1.05

### The Effect of the Intervention on Red and Processed Meat Consumption

**FIGURE 1:**
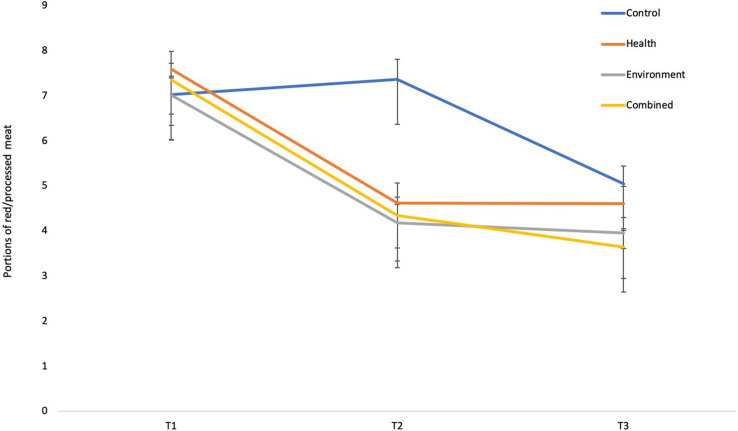
Average red and processed meat consumption across time. Error bars represent standard error ± mean.

Participants’ average reported consumption of red and processed meat is summarized in Figure 1. The results from the linear-mixed model showed that there was no significant main effect of condition when controlling for time (see Table 2). There was, however, a significant interaction between time and condition, whereby participants in the environment, health and combined conditions significantly reduced their red and processed meat consumption at T2 compared to T1, while participants in the control condition showed no change in consumption. There was a significant main effect of time when controlling for condition, whereby participants in all conditions significantly reduced their consumption of red and processed meat at T3 compared to T1. The results also showed a significant interaction between time and condition, whereby participants in the combined condition reduced their red and processed meat consumption significantly more than control participants at T3 compared to T1. There were no other interaction effects. Thus, the results showed that providing information on the health and/or environmental impacts of meat had a significant effect on reducing red and processed meat consumption during the intervention and one-month later, supporting Hypothesis 1.

**TABLE 2:** Multi-level model regression coefficients for red and processed meat.

**Predictors**	**Estimate**	**Standard Error**	**df**	***t***	***p***	**95% CI**
Intercept	7.03	0.40	334.36	17.43	0.000**	[6.23,7.82]
**Time**						
Time 2	0.34	0.53	388.11	0.64	0.522	[−0.70,1.38]
Time 3	–1.98	0.49	363.65	–4.09	0.000**	[−2.94,−1.03]
**Condition**						
Health	0.56	0.56	334.36	1.00	0.317	[−0.54,1.67]
Environment	–0.02	0.55	334.36	–0.03	0.978	[−1.10,1.07]
Combined	0.32	0.55	334.36	0.59	0.558	[−0.76,1.40]
**Interactions**						
Time 2 × Health	–3.31	0.74	388.11	–4.47	0.000**	[−4.76,−1.85]
Time 2 × Environment	–3.17	0.72	387.38	–4.41	0.000**	[−4.59,−1.76]
Time 2 × Combined	–3.35	0.71	387.52	–4.69	0.000**	[−4.75,−1.95]
Time 3 × Health	–1.00	0.68	363.80	–1.48	0.140	[−2.32,0.33]
Time 3 × Environment	–1.08	0.65	360.59	–1.65	0.100	[−2.37,0.21]
Time 3 × Combined	–1.72	0.66	362.58	–2.62	0.009*	[−3.01,−0.43]

*^∗^*p* < 0.01, ^∗∗^*p* < 0.001.*

The mean differences in red and processed meat consumption reported by participants at T2 compared to T1, and at T3 compared to T1, were calculated and compared across the different experimental conditions to assess whether there were any significant differences between the health, environment and combined conditions on reducing participants’ red and processed meat consumption. Interpretation of the confidence intervals showed that there were no significant differences between the environment and health condition (M difference = −0.14, CI = −1.16,0.88), the health and combined condition (M difference = 0.04, CI = −1.11,1.19) or the environment and combined condition (M difference = 0.18, CI = −0.82,1.17) in reducing red and processed meat consumption at T2. There were also no significant differences between the environment and health condition (M difference = −0.06, CI = −1.03,0.91), the health and combined condition (M difference = 0.38, CI = −0.62,1.36), or the environment and combined condition (M difference = 0.43, CI = −0.48,1.35), in reducing red and processed meat consumption at T3. Thus, there were no significant differences in the amount red and processed meat reduced by participants in the environment, health and combined conditions at either time point.

### Investigating Behavioral Spillover

Tables showing regression parameters for all of the spillover analyses can be viewed in the Supplementary Material.

### Positive Spillover Following Red and Processed Meat Reduction

**FIGURE 2:**
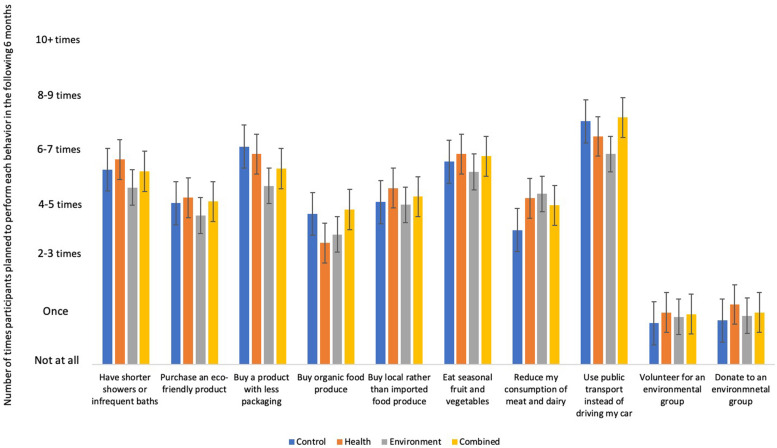
Participants average willingness to perform pro-environmental behaviors at time 2.

**FIGURE 3:**
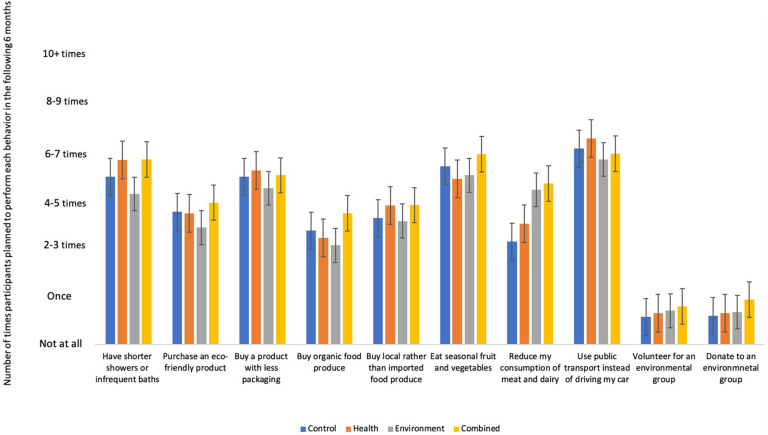
Participants average willingness to perform pro-environmental behaviors at time 3.

Participants’ willingness to perform each of the pro-environmental behaviors measured at times T2 and T3 is summarized in Figures 2, 3, respectively. Multiple linear regressions were conducted to investigate whether reduced consumption of red and processed meat increased participants’ willingness to perform other pro-environmental behaviors at T2 and T3, respectively. Differences between the experimental and control conditions were also investigated using dummy coded variables. Analysis was first conducted to investigate behavioral spillover at T2. Multiple linear regressions were conducted with change in red and processed meat consumption (T2–T1) and dummy coded environment, health and combined conditions, with the control condition as the reference group, as independent variables. Willingness to perform each of the pro-environmental behaviors measured at T2 were the dependent variables. The model did not significantly predict participants’ willingness to perform any of the pro-environmental behaviors measured at T2 after correcting for multiple comparisons.

Multiple linear regressions were then conducted with change in red and processed meat consumption (T3–T1) and dummy coded environment, health and combined conditions, with the control condition as the reference group, as independent variables. Willingness to engage in each of the pro-environmental behaviors measured at T3 were the dependent variables. The model significantly predicted participants’ willingness to eat less meat and dairy products [*F*(4,228) = 5.35, *p* < 0.001). A reduced consumption of red and processed meat was associated with an increased willingness to eat less meat and dairy (*B* = −0.78, *p* = 0.018, adjusted *R*^2^ = 0.07). The results also showed a significant difference where participants in the environment and combined condition were significantly more willing to reduce their meat and dairy compared to participants in the control condition (*B* = 1.00, *p* = 0.006; *B* = 1.07, *p* = 0.004, respectively). There was no significant difference between the health and control condition (*p* = 0.491). When the reference group was switched, the results showed that participants in the combined and environmental conditions were also significantly more willing to eat less meat and dairy compared to participants in the health condition (*B* = 0.81, *p* = 0.022; *B* = 0.74, *p* = 0.032, respectively). There was no significant difference between the combined and environment condition (*B* = 0.07, *p* = 0.841). The model did not significantly predict any other pro-environmental behaviors at T3 after correcting for multiple comparisons. Thus, there was some evidence to support that a reduced consumption of red and processed meat one-month after the intervention led to an increased willingness to reduce ones’ meat and dairy consumption. However, a reduced consumption of red and processed meat did not predict any other, untargeted, pro-environmental behaviors at either time point. Thus, Hypothesis 2 was only partially supported.

### Pro-environmental Identity as a Driver of Spillover

Participants’ pro-environmental identity was just above the midpoint at T1 (M = 4.54, SD = 1.04), T2 (M = 4.64, SD = 1.00) and T3 (M = 4.69, SD = 1.00), with little variation across these three timepoints. Following this, paired-samples *t*-tests showed that pro-environmental identity did not significantly increase at T2 [*t*(248) = −1.49, *p* = 0.139] or T3 [*t*(233) = −1.58, *p* = 0.116] compared to T1. Thus, the intervention did not appear to affect participants’ pro-environmental identity.

Hierarchical multiple regressions were conducted to investigate whether reduced consumption of red and processed meat would predict increased pro-environmental identity at time T2. Pro-environmental identity at T1 was included as a covariate, given that the extent to which a participant is able to increase their identity after the intervention is dependent on their initial pro-environmental identity at baseline. Pro-environmental identity at T1 was entered in block 1, change in red and processed meat consumption (T2–T1) and dummy coded variables for the environment, health and combined conditions were entered in block 2, with the control condition as the reference group. Change in pro-environmental identity (T2–T1) was the dependent variable. The results showed that the overall model was significant [*F*(5,242) = 9.91, *p* = < 0.001, adjusted *R*^2^ = 0.15]. Baseline pro-environmental identity explained 17% of variance in block 1 (*R*^2^ = 0.17). However, adding change in red and processed meat consumption with the dummy coded conditions in block 2 did not explain any additional variance and did not significantly improve the model [*R*^2^ change = 0.00, *F* change (4,242) = 0.36, *p* = 0.837]. In the overall model, change in red and processed meat consumption was not a significant predicter of change in pro-environmental identity when controlling for baseline identity. Participants in the environment, health and combined conditions did not show a greater change in their pro-environmental identity compared to participants in the control condition.

The above analysis was repeated to investigate whether reduced consumption of red and processed meat would predict increased pro-environmental identity at time T3. As with the above analysis, pro-environmental identity at T1 was entered in block 1, change in red and processed meat consumption (T3−T1) and dummy coded variables for the environment, health and combined condition were entered in block 2, with the control condition as the reference group. Change in pro-environmental identity (T3–T1) was the dependent variable. The results showed that the overall model was significant [*F*(5,231) = 13.66, *p* = < 0.001, adjusted *R*^2^ = 2.11]. Baseline pro-environmental identity explained 22% of variance in block 1 (*R*^2^ = 0.22). However, adding change in red and processed meat consumption with the dummy coded conditions in block 2 explained only any additional 1% of variance and did not significantly improve the model [*R*^2^ change = 0.01, *F* change (4,231) = 1.02, *p* = 0.399]. In the overall model, change in red and processed meat consumption was not a significant predicter of change in pro-environmental identity when controlling for baseline identity. Participants in the environment, health and combined conditions did not show a greater change in their pro-environmental identity compared to participants in the control condition. Thus, the results did not show any evidence that participants’ reduced consumption of red and processed meat was associated with increased pro-environmental identity shortly after the intervention or one-month later, meaning Hypothesis 3 was not supported.

Multiple regressions were subsequently conducted to investigate whether increased pro-environmental identity would predict increased willingness to perform pro-environmental behaviors at times T2 and T3, respectively. Change in pro-environmental identity at T2 (compared to T1) with dummy coded variables for the environment, health and combined conditions, with the control condition as the reference group, were entered as predictors of participants’ willingness to perform each of the 10 pro-environmental behaviors measured at T2. The model was not significant for any of the pro-environmental behaviors measured at T2 (all *p’*s > 0.05).

The above analysis was repeated with change in pro-environmental identity at T3 (compared to T1) and with dummy coded variables for the environment, health and combined conditions, with the control condition as the reference group, entered as predictors of participants’ willingness to perform each of the 10 pro-environmental behaviors measured at T3. The results showed that the model did not significantly predict any of the pro-environmental behaviors at T3 after correcting for multiple comparisons. Thus, there was no evidence to suggest that increased pro-environmental identity lead to an increased willingness to engage in additional pro-environmental behaviors, shortly after the intervention or one-month later, meaning Hypothesis 4 was not supported.

## Discussion

This study investigated whether providing information about the environmental and/or health impacts of eating meat would reduce participants’ red and processed meat consumption and encourage additional untargeted pro-environmental behaviors. First, the results showed that providing information on the environmental and/or health impacts of meat was effective in significantly reducing participants’ red and processed meat consumption during the intervention and one-month later, supporting Hypothesis 1. This adds to a growing body of literature investigating the effectiveness of informational strategies on encouraging meat reduction (e.g., see Bianchi et al., 2018; Harguess et al., 2019). Specifically, this study shows that providing information on the different impacts of meat can be an effective strategy for reducing red and processed meat consumption when this is also paired with a clear time-oriented goal. This study builds on past literature which has tended to focus on the effect of interventions on changing attitudes or intentions (ibid), by demonstrating the effectiveness of information provision on eliciting behavior change over a prolonged period of time. While past literature has demonstrated that providing health (e.g., Berndsen and Van Der Pligt, 2005; Cordts et al., 2014; Bertolotti et al., 2019), and to a lesser extent environmental (e.g., Hunter and Röös, 2016; Graham and Abrahamse, 2017; Stea and Pickering, 2019) messages can be effective in reducing meat consumption, the evidence on combined health and environmental messages has been mixed (e.g., Amiot et al., 2018; Carfora et al., 2019b). We add to this literature by demonstrating the effectiveness of combined messages in reducing red and processed meat consumption in the current study. Furthermore, only participants who received information on the combined impacts of red and processed meat reduced their red and processed meat significantly more than control participants at T3. Thus, in some cases the combined messages had an even stronger effect on reducing red and processed meat consumption compared to providing information on the health and environmental impacts only. This supports the notion that drawing on multiple motives can be an effective strategy to encourage a reduced consumption of meat, compared to focusing on different motives in isolation (De Boer et al., 2013).

The fact that participants in the control condition also reduced their red and processed meat consumption suggests that some aspect of the intervention other than information provision may have led participants to reduce their meat consumption. One possible explanation is that completing the daily food diaries led control participants to monitor their meat intake, causing a reduced consumption of red and processed meat. Previous literature has supported the role of self-monitoring in contributing to meat reduction (e.g., Carfora et al., 2017). Furthermore, past research has also demonstrated a similar delayed effect of an intervention containing a self-monitoring aspect on reducing red meat consumption (Amiot et al., 2018). Thus, it is possible that completing the daily food diaries lead to a delayed effect of self-monitoring on reducing red and processed meat consumption for participants in the control condition. This would indicate that providing information on the health and/or environmental impacts of meat and encouraging self-monitoring, could be an effective strategy for reducing excess meat consumption over a prolonged period of time. However, this explanation is speculative and would need to be validated by further research. An alternative explanation is that participants from different conditions shared information about the study aims in the delay between the intervention and the one-month follow-up, which could have led control participants to reduce their consumption as a result of social desirability. This possibility cannot be ruled out, as many participants were studying on the same course and therefore may have been acquainted with each other.

Second, the results suggested some limited evidence of behavioral spillover, partially supporting hypothesis 2. After correcting for multiple comparisons, there was only a significant effect where a reduced consumption of red and processed meat was associated with an increased willingness to eat less meat and dairy. We view this as partial evidence of spillover, considering the similarity between reducing ones’ red and processed meat consumption and reducing ones’ meat and dairy consumption. Nevertheless, this is a promising finding which suggests that reducing ones’ red and processed meat consumption has the potential to encourage further dietary change. It is interesting to note that participants in the environmental and combined conditions were significantly more willing to perform this behavior than those in the control and health conditions. This suggests that providing information on the environmental and the combined environmental and health impacts of meat was particularly effective in encouraging further dietary change, compared to providing information only on the health impacts of meat. These findings contribute to literature investigating the effectiveness of combined messaging to encourage pro-environmental behavior, which has shown that highlighting financial motivations can reduce the effectiveness of pro-environmental messages to encourage pro-environmental behavior (e.g., Schwartz et al., 2015). On the other hand, the current study demonstrates that combining health with environmental motives can promote pro-environmental behavior and have longer lasting effects on behavior than when this information is communicated separately.

Third, the results showed that the intervention did not have any significant effects on pro-environmental identity and that pro-environmental identity did not have any significant effect on participants’ willingness to engage in different pro-environmental behaviors, meaning Hypotheses 3 and 4 were not supported. One possible explanation for these findings is that reducing one’s meat consumption is not necessarily an environmentally salient behavior, given that many people are not aware of the negative environmental impacts associated with meat (e.g., Bailey et al., 2014; Macdiarmid et al., 2016). This could also explain the lack of evidence for behavioral spillover for untargeted pro-environmental behaviors in the current study, given that an increased pro-environmental identity can act as a catalyst for positive spillover (e.g., Cornelissen et al., 2008; Van der Werff et al., 2013). Future research could therefore focus on increasing the saliency of meat reduction as a pro-environmental behavior, to promote pro-environmental identity and subsequent spillover effects. This is supported by recent evidence in which participants were found to show a stronger pro-environmental identity and an increased uptake of different pro-environmental behaviors following a workplace intervention, which focused specifically and exclusively on the environmental impacts of meat (Verfuerth et al., 2019). Although this study showed limited evidence of positive spillover, it is worth noting that the results also did not show any evidence of negative behavioral spillover. This is an encouraging finding, demonstrating that our intervention successfully reduced participants’ red and processed meat consumption, without inadvertently increasing negative environmental impacts through moral licensing or contribution ethic, as observed in other pro-environmental behavior change interventions (e.g., Tiefenbeck et al., 2013).

### Limitations and Future Directions

It is worth noting that there are some limitations of the current study. First, the measure of red and processed meat consumption required participants to indicate the number of servings of red and processed meat they had eaten in the previous week. Although participants were provided with example portion sizes for red and processed meat, this might not have been sufficient to ensure a precise measure participants’ meat consumption. Participants also may not have been able to accurately recall the amount of red and processed meat they had consumed retrospectively, during the previous week. On the other hand, using food diaries throughout the study duration might have provided a more accurate representation of participants’ red and processed meat consumption, as food diaries would allow for food choices to be reported on a day-to-day basis and with different response options for different serving sizes. That the food diaries were completed only during the two-week intervention period is a limitation of this study, as the diary data could not be compared from before to after the intervention. Future studies would benefit from implementing food diaries across all study timepoints, to enhance the accuracy of self-report measures of meat consumption. Alternatively, future research might benefit from using more objective measures of meat consumption, for example by collecting shopping receipts (e.g., Kaiser et al., 2020), to overcome potential issues associated with self-report data, such as false reporting and desirability effects. Second this study investigated the effectiveness of different messages in reducing red and processed meat consumption, without measuring whether participants subsequently increased their consumption of other plant-based foods. It is therefore not possible to determine whether participants simply reduced their consumption of meat and thus overall food consumption, which could be considered a form of dieting. Although there is recent literature investigating the consumption of alternatives to meat, such as plant-based alternatives, insects and vegetarian meals (e.g., Schösler et al., 2012; Verbeke, 2015; Hartmann and Siegrist, 2017; Gómez-Luciano et al., 2019) studies have not tended to investigate whether these foods might be chosen as replacements for meat during, or after, an intervention aimed at reducing meat consumption. That this is not addressed in the current study is a limitation that should be considered in future research, to establish whether individuals are able to adopt a diet that is healthy and can realistically be maintained following a reduced consumption of red and processed meat. Third, only two of the ten measured pro-environmental behaviors were public-sphere behaviors, limiting the likelihood of detecting potential public-sphere spillover effects. Future research should investigate the potential for positive spillover from meat-reduction to public-sphere pro-environmental behaviors more extensively, given that public-sphere behaviors such as active political engagement, environmental lobbying and support for environmental policies could have a greater positive environmental impact compared to private-sphere behaviors, such as recycling or buying eco-friendly products (e.g., Thøgersen and Crompton, 2009; Lauren et al., 2017). Fourth, participants indicated their intentions to perform different pro-environmental behaviors in the upcoming months. However, there is often a gap between people’s intentions and actions (e.g., Hassan et al., 2016). Future research might therefore benefit from investigating spillover using observable measures of behavior to improve the accuracy of this measure. Finally, the reliance on a student sample means that the findings may not be generalisable to the wider public. Thus, future research might benefit from using different participant samples, for example members of the general public, to improve generalisability.

## Conclusion

This study contributes to the emerging literature on strategies aimed at encouraging a reduced meat consumption by demonstrating the effectiveness of information provision on reducing red and processed meat consumption and potentially spilling over to other dietary changes. These findings contribute to a greater understanding of the potential effectiveness of different strategies aimed at reducing meat consumption and highlight the usefulness of health and/or environmental messages in promoting healthier more sustainable diets, with no apparent negative impact on other pro-environmental lifestyle choices.

## Data Availability Statement

The raw data supporting the conclusion of this article will be made available by the authors, without undue reservation.

## Ethics Statement

This study was reviewed and approved by the Cardiff University School of Psychology Research Ethics Committee. The participants provided their written informed consent to participate in this study.

## Author Contributions

EW conducted the research, analyzed the data, and drafted the manuscript. WP and LW advised on design, analysis and interpretation, and provided comments and edits to the manuscript. All authors contributed to the article and approved the submitted version.

## Conflict of Interest

The authors declare that the research was conducted in the absence of any commercial or financial relationships that could be construed as a potential conflict of interest.
