# Supplementary material for: Two Birds, One Stone: The Effectiveness of Health and Environmental Messages to Reduce Meat Consumption and Encourage Pro-environmental Behavioral Spillover
Source: Front Psychol. 2020 Oct 7;11:577111. doi: 10.3389/fpsyg.2020.577111 (PMC7575709; doi:10.3389/fpsyg.2020.577111)
Supplement: Supplementary file 1 [file Table_1.DOCX]

Supplementary Material

**Supplementary Methods**

Appendix S1. Information presented to participants before the randomised messaging intervention

**Control:**

For the next part of the study, you will be asked to record all of the food you consume by keeping a daily food diary for 14 consecutive days. You will be sent some information including a reminder and a link to the food diary each day via the private chat on Facebook messenger during this time.

**We ask that you do not change your diet in anyway during the study period.**

**Health:**

For the next part of the study, you will be asked to record all of the food you consume by keeping a daily food diary for 14 consecutive days. You will be sent some information including a reminder and a link to the food diary each day via the private chat on Facebook messenger during this time.

**During the study period we ask that you try to eat no more than two medium portions of red (including processed) meat each week.** This is because several scientific studies have shown that red/processed meat negatively affects health through an increased risk of chronic diseases including bowel, respiratory, kidney, liver and heart disease, as well as diabetes and stroke. Furthermore, red/processed meat consumption is associated with several forms of cancer, including stomach, pancreatic and bowel cancer, in addition to obesity, metabolic syndrome and joint problems. This is thought to result from the presence of harmful substances in red/processed meat and other chemicals produced through cooking. Therefore, reducing your red/processed meat consumption will protect your health.

**Environment:**

For the next part of the study, you will be asked to record all of the food you consume by keeping a daily food diary for 14 consecutive days. You will be sent some information including a reminder and a link to the food diary each day via the private chat on Facebook messenger during this time.

**During the study period we ask that you try to eat no more than two medium portions of red (including processed) meat each week.** This is because several scientific studies have shown that red/processed meat production negatively affects the environment through the release of harmful greenhouse gases, driving climate change. Furthermore, red/processed meat production is associated with harmful changes to land, water and air, including deforestation, soil acidification, desertification, biodiversity loss, water pollution, and air pollution. This results from the excessive amount of land, water and fertilizer that is needed to rear livestock for red/processed meat consumption. Therefore, reducing your red/processed meat consumption will protect the environment from the harmful effects of its production.

**Combined:**

For the next part of the study, you will be asked to record all of the food you consume by keeping a daily food diary for 14 consecutive days. You will be sent some information including a reminder and a link to the food diary each day via the private chat on Facebook messenger during this time.

**During the study period we ask that you try to eat no more than two medium portions of red (including processed) meat each week.**This is because several scientific studies have shown that red/processed meat consumption is linked to a series of negative health and environmental outcomes.

Red/processed meat negatively affects health through an increased risk of chronic diseases including bowel, respiratory, kidney, liver and heart disease, as well as diabetes and stroke. Furthermore, red/processed meat consumption is associated with several forms of cancer, including stomach, pancreatic and bowel cancer, in addition to obesity, metabolic syndrome and joint problems. This is thought to result from the presence of harmful substances in red/processed meat and other chemicals produced through cooking. Red/processed meat production negatively affects the environment through the release of harmful greenhouse gases, driving climate change. Furthermore, red/processed meat production is associated with harmful changes to land, water and air, including deforestation, soil acidification, desertification, biodiversity loss, water pollution, and air pollution. This results from the excessive amount of land, water and fertilizer that is needed to rear livestock for red/processed meat consumption. Therefore, reducing your  red/processed meat consumption will protect both the environment and your health.

Appendix S2. Messages sent to participants each day of the randomised messaging intervention

| **Control** | **Health** | **Environment** | **Combined** |
| --- | --- | --- | --- |
| MORNING:  A link to today’s food diary will be sent to you this evening so that you can record your food intake for today.  That is all for now. I will write to you tonight with further instructions.  EVENING:  AFTER DINNER, please remember to record all of the food you eat today using today’s food diary.  Click “Go to Diary” to go to today’s food diary. Please make sure you press the red arrow at the end of the diary to save your responses. | MORNING:  If you eat only a small amount of red and processed meat, you will protect your health by reducing the likelihood of developing cancer.  Remember to try and eat no more than two portions of red/processed meat this week.  A link to today’s food diary will be sent to you this evening so that you can record your food intake for today.  EVENING:  If you eat only a small amount of red and processed meat, you will protect your health by reducing the likelihood of developing cancer.  Remember to try and eat no more than two portions of red/processed meat this week.  AFTER DINNER, please remember to record all of the food you eat today using today’s food diary.  Click “Go to Diary” to go to today’s food diary. Please make sure you press the red arrow at the end of the diary to save your responses. | MORNING:  If you eat only a small amount of red and processed meat, you will protect the environment by reducing the release of harmful greenhouse gases.  Remember to try and eat no more than two portions of red/processed meat this week.  A link to today’s food diary will be sent to you this evening so that you can record your food intake for today.  EVENING:  If you eat only a small amount of red and processed meat, you will protect the environment by reducing the release of harmful greenhouse gases.  Remember to try and eat no more than two portions of red/processed meat this week.  AFTER DINNER, please remember to record all of the food you eat today using today’s food diary.  Click “Go to Diary” to go to today’s food diary. Please make sure you press the red arrow at the end of the diary to save your responses. | MORNING:  If you eat only a small amount of red and processed meat, you will protect the environment from harmful greenhouse gases and you will protect your health by reducing the likelihood of developing cancer.  Remember to try and eat no more than two portions of red/processed meat this week.  A link to today’s food diary will be sent to you this evening so that you can record your food intake for today.  EVENING:  If you eat only a small amount of red and processed meat, you will protect the environment from harmful greenhouse gases and you will protect your health by reducing the likelihood of developing cancer.  Remember to try and eat no more than two portions of red/processed meat this week.  AFTER DINNER, please remember to record all of the food you eat today using today’s food diary.  Click “Go to Diary” to go to today’s food diary. Please make sure you press the red arrow at the end of the diary to save your responses. |
| MORNING:  A link to today’s food diary will be sent to you this evening so that you can record your food intake for today.  That is all for now. I will write to you tonight with further instructions.  EVENING:  AFTER DINNER, please remember to record all of the food you eat today using today’s food diary.  Click “Go to Diary” to go to today’s food diary. Please make sure you press the red arrow at the end of the diary to save your responses. | MORNING:  If you eat only a small amount of red and processed meat, you will protect your health by reducing the likelihood of becoming obese.  Remember to try and eat no more than two portions of red/processed meat this week.  A link to today’s food diary will be sent to you this evening so that you can record your food intake for today.  EVENING:  If you eat only a small amount of red and processed meat, you will protect your health by reducing the likelihood of becoming obese.  Remember to try and eat no more than two portions of red/processed meat this week.  AFTER DINNER, please remember to record all of the food you eat today using today’s food diary.  Click “Go to Diary” to go to today’s food diary. Please make sure you press the red arrow at the end of the diary to save your responses. | MORNING:  If you eat only a small amount of red and processed meat, you will protect the environment by reducing the amount of deforestation.  Remember to try and eat no more than two portions of red/processed meat this week.  A link to today’s food diary will be sent to you this evening so that you can record your food intake for today.  EVENING:  If you eat only a small amount of red and processed meat, you will protect the environment by reducing the amount of deforestation.  Remember to try and eat no more than two portions of red/processed meat this week.  AFTER DINNER, please remember to record all of the food you eat today using today’s food diary.  Click “Go to Diary” to go to today’s food diary. Please make sure you press the red arrow at the end of the diary to save your responses. | MORNING:  If you eat only a small amount of red and processed meat, you will protect your health by reducing the likelihood of becoming obese and you will protect the environment by reducing the amount of deforestation.  Remember to try and eat no more than two portions of red/processed meat this week.  A link to today’s food diary will be sent to you this evening so that you can record your food intake for today.  EVENING:  If you eat only a small amount of red and processed meat, you will protect your health by reducing the likelihood of becoming obese and you will protect the environment by reducing the amount of deforestation.  Remember to try and eat no more than two portions of red/processed meat this week.  AFTER DINNER, please remember to record all of the food you eat today using today’s food diary.  Click “Go to Diary” to go to today’s food diary. Please make sure you press the red arrow at the end of the diary to save your responses. |
| MORNING:  A link to today’s food diary will be sent to you this evening so that you can record your food intake for today.  That is all for now. I will write to you tonight with further instructions.  EVENING:  AFTER DINNER, please remember to record all of the food you eat today using today’s food diary.  Click “Go to Diary” to go to today’s food diary. Please make sure you press the red arrow at the end of the diary to save your responses. | MORNING:  If you eat only a small amount of red and processed meat, you will protect your health by reducing the likelihood of developing heart disease.  Remember to try and eat no more than two portions of red/processed meat this week.  A link to today’s food diary will be sent to you this evening so that you can record your food intake for today.  EVENING:  If you eat only a small amount of red and processed meat, you will protect your health by reducing the likelihood of developing heart disease.  Remember to try and eat no more than two portions of red/processed meat this week.  AFTER DINNER, please remember to record all of the food you eat today using today’s food diary.  Click “Go to Diary” to go to today’s food diary. Please make sure you press the red arrow at the end of the diary to save your responses. | MORNING:  If you eat only a small amount of red and processed meat, you will protect the environment by reducing water waste.  Remember to try and eat no more than two portions of red/processed meat this week.  A link to today’s food diary will be sent to you this evening so that you can record your food intake for today.  EVENING:  If you eat only a small amount of red and processed meat, you will protect the environment by reducing water waste.  Remember to try and eat no more than two portions of red/processed meat this week.  AFTER DINNER, please remember to record all of the food you eat today using today’s food diary.  Click “Go to Diary” to go to today’s food diary. Please make sure you press the red arrow at the end of the diary to save your responses. | MORNING:  If you eat only a small amount of red and processed meat, you will protect the environment by reducing water waste and you will protect your health by reducing the likelihood of developing heart disease.  Remember to try and eat no more than two portions of red/processed meat this week.  A link to today’s food diary will be sent to you this evening so that you can record your food intake for today.  EVENING:  If you eat only a small amount of red and processed meat, you will protect the environment by reducing water waste and you will protect your health by reducing the likelihood of developing heart disease.  Remember to try and eat no more than two portions of red/processed meat this week.  AFTER DINNER, please remember to record all of the food you eat today using today’s food diary.  Click “Go to Diary” to go to today’s food diary. Please make sure you press the red arrow at the end of the diary to save your responses. |
| MORNING:  A link to today’s food diary will be sent to you this evening so that you can record your food intake for today.  That is all for now. I will write to you tonight with further instructions.  EVENING:  AFTER DINNER, please remember to record all of the food you eat today using today’s food diary.  Click “Go to Diary” to go to today’s food diary. Please make sure you press the red arrow at the end of the diary to save your responses. | MORNING:  If you eat only a small amount of red and processed meat, you will protect your health by reducing the likelihood of becoming diabetic.  Remember to try and eat no more than two portions of red/processed meat this week.  A link to today’s food diary will be sent to you this evening so that you can record your food intake for today.  EVENING:  If you eat only a small amount of red and processed meat, you will protect your health by reducing the likelihood of becoming diabetic.  Remember to try and eat no more than two portions of red/processed meat this week.  AFTER DINNER, please remember to record all of the food you eat today using today’s food diary.  Click “Go to Diary” to go to today’s food diary. Please make sure you press the red arrow at the end of the diary to save your responses. | MORNING:  If you eat only a small amount of red and processed meat, you will protect the environment by reducing water pollution.  Remember to try and eat no more than two portions of red/processed meat this week.  A link to today’s food diary will be sent to you this evening so that you can record your food intake for today.  EVENING:  If you eat only a small amount of red and processed meat, you will protect the environment by reducing water pollution.  Remember to try and eat no more than two portions of red/processed meat this week.  AFTER DINNER, please remember to record all of the food you eat today using today’s food diary.  Click “Go to Diary” to go to today’s food diary. Please make sure you press the red arrow at the end of the diary to save your responses. | MORNING:  If you eat only a small amount of red and processed meat, you will protect your health by reducing the likelihood of becoming diabetic and you will protect the environment by reducing water pollution.  Remember to try and eat no more than two portions of red/processed meat this week.  A link to today’s food diary will be sent to you this evening so that you can record your food intake for today.  _______________________________________________  EVENING:  If you eat only a small amount of red and processed meat, you will protect your health by reducing the likelihood of becoming diabetic and you will protect the environment by reducing water pollution.  Remember to try and eat no more than two portions of red/processed meat this week.  AFTER DINNER, please remember to record all of the food you eat today using today’s food diary.  Click “Go to Diary” to go to today’s food diary. Please make sure you press the red arrow at the end of the diary to save your responses. |
| MORNING:  A link to today’s food diary will be sent to you this evening so that you can record your food intake for today.  That is all for now. I will write to you tonight with further instructions.  EVENING:  AFTER DINNER, please remember to record all of the food you eat today using today’s food diary.  Click “Go to Diary” to go to today’s food diary. Please make sure you press the red arrow at the end of the diary to save your responses. | MORNING:  If you eat only a small amount of red and processed meat, you will protect your health by reducing the likelihood of developing colon cancer.  Remember to try and eat no more than two portions of red/processed meat this week.  A link to today’s food diary will be sent to you this evening so that you can record your food intake for today.  EVENING:  If you eat only a small amount of red and processed meat, you will protect your health by reducing the likelihood of developing colon cancer.  Remember to try and eat no more than two portions of red/processed meat this week.  AFTER DINNER, please remember to record all of the food you eat today using today’s food diary.  Click “Go to Diary” to go to today’s food diary. Please make sure you press the red arrow at the end of the diary to save your responses. | MORNING:  If you eat only a small amount of red and processed meat, you will protect the environment by reducing excessive land use.  Remember to try and eat no more than two portions of red/processed meat this week.  A link to today’s food diary will be sent to you this evening so that you can record your food intake for today.  EVENING:  If you eat only a small amount of red and processed meat, you will protect the environment by reducing excessive land use.  Remember to try and eat no more than two portions of red/processed meat this week.  AFTER DINNER, please remember to record all of the food you eat today using today’s food diary.  Click “Go to Diary” to go to today’s food diary. Please make sure you press the red arrow at the end of the diary to save your responses. | MORNING:  If you eat only a small amount of red and processed meat, you will protect the environment by reducing excessive land use and you will protect your health by reducing the likelihood of developing colon cancer.  Remember to try and eat no more than two portions of red/processed meat this week.  A link to today’s food diary will be sent to you this evening so that you can record your food intake for today.  EVENING:  If you eat only a small amount of red and processed meat, you will protect the environment by reducing excessive land use and you will protect your health by reducing the likelihood of developing colon cancer.  Remember to try and eat no more than two portions of red/processed meat this week.  AFTER DINNER, please remember to record all of the food you eat today using today’s food diary.  Click “Go to Diary” to go to today’s food diary. Please make sure you press the red arrow at the end of the diary to save your responses. |
| MORNING:  A link to today’s food diary will be sent to you this evening so that you can record your food intake for today.  That is all for now. I will write to you tonight with further instructions.  EVENING:  AFTER DINNER, please remember to record all of the food you eat today using today’s food diary.  Click “Go to Diary” to go to today’s food diary. Please make sure you press the red arrow at the end of the diary to save your responses. | MORNING:  If you eat only a small amount of red and processed meat, you will protect your health by reducing the likelihood of developing bowel cancer.  Remember to try and eat no more than two portions of red/processed meat this week.  A link to today’s food diary will be sent to you this evening so that you can record your food intake for today.  EVENING:  If you eat only a small amount of red and processed meat, you will protect your health by reducing the likelihood of developing bowel cancer.  Remember to try and eat no more than two portions of red/processed meat this week.  AFTER DINNER, please remember to record all of the food you eat today using today’s food diary.  Click “Go to Diary” to go to today’s food diary. Please make sure you press the red arrow at the end of the diary to save your responses. | MORNING:  If you eat only a small amount of red and processed meat, you will protect the environment by reducing excessive fertilizer use.  Remember to try and eat no more than two portions of red/processed meat this week.  A link to today’s food diary will be sent to you this evening so that you can record your food intake for today.  EVENING:  If you eat only a small amount of red and processed meat, you will protect the environment by reducing excessive fertilizer use.  Remember to try and eat no more than two portions of red/processed meat this week.  AFTER DINNER, please remember to record all of the food you eat today using today’s food diary.  Click “Go to Diary” to go to today’s food diary. Please make sure you press the red arrow at the end of the diary to save your responses. | MORNING:  If you eat only a small amount of red and processed meat, you will protect your health by reducing the likelihood of developing bowel cancer and you will protect the environment by reducing excessive fertilizer use.  Remember to try and eat no more than two portions of red/processed meat this week.  A link to today’s food diary will be sent to you this evening so that you can record your food intake for today.  EVENING:  If you eat only a small amount of red and processed meat, you will protect your health by reducing the likelihood of developing bowel cancer and you will protect the environment by reducing excessive fertilizer use.  Remember to try and eat no more than two portions of red/processed meat this week.  AFTER DINNER, please remember to record all of the food you eat today using today’s food diary.  Click “Go to Diary” to go to today’s food diary. Please make sure you press the red arrow at the end of the diary to save your responses. |
| MORNING:  A link to today’s food diary will be sent to you this evening so that you can record your food intake for today.  That is all for now. I will write to you tonight with further instructions.  EVENING:  AFTER DINNER, please remember to record all of the food you eat today using today’s food diary.  Click “Go to Diary” to go to today’s food diary. Please make sure you press the red arrow at the end of the diary to save your responses. | MORNING:  If you eat only a small amount of red and processed meat, you will protect your health by reducing the likelihood of developing metabolic syndrome.  Remember to try and eat no more than two portions of red/processed meat this week.  A link to today’s food diary will be sent to you this evening so that you can record your food intake for today.  EVENING:  If you eat only a small amount of red and processed meat, you will protect your health by reducing the likelihood of developing metabolic syndrome.  Remember to try and eat no more than two portions of red/processed meat this week.  AFTER DINNER, please remember to record all of the food you eat today using today’s food diary.  Click “Go to Diary” to go to today’s food diary. Please make sure you press the red arrow at the end of the diary to save your responses. | MORNING:  If you eat only a small amount of red and processed meat, you will protect the environment by reducing biodiversity loss.  Remember to try and eat no more than two portions of red/processed meat this week.  A link to today’s food diary will be sent to you this evening so that you can record your food intake for today.  EVENING:  If you eat only a small amount of red and processed meat, you will protect the environment by reducing biodiversity loss.  Remember to try and eat no more than two portions of red/processed meat this week.  AFTER DINNER, please remember to record all of the food you eat today using today’s food diary.  Click “Go to Diary” to go to today’s food diary. Please make sure you press the red arrow at the end of the diary to save your responses. | MORNING:  If you eat only a small amount of red and processed meat, you will protect the environment by reducing biodiversity loss and you will protect your health by reducing the likelihood of developing metabolic syndrome.  Remember to try and eat no more than two portions of red/processed meat this week.  A link to today’s food diary will be sent to you this evening so that you can record your food intake for today.  EVENING:  If you eat only a small amount of red and processed meat, you will protect the environment by reducing biodiversity loss and you will protect your health by reducing the likelihood of developing metabolic syndrome.  Remember to try and eat no more than two portions of red/processed meat this week.  AFTER DINNER, please remember to record all of the food you eat today using today’s food diary.  Click “Go to Diary” to go to today’s food diary. Please make sure you press the red arrow at the end of the diary to save your responses. |
| MORNING:  A link to today’s food diary will be sent to you this evening so that you can record your food intake for today.  That is all for now. I will write to you tonight with further instructions.  EVENING:  AFTER DINNER, please remember to record all of the food you eat today using today’s food diary.  Click “Go to Diary” to go to today’s food diary. Please make sure you press the red arrow at the end of the diary to save your responses. | MORNING:  If you eat only a small amount of red and processed meat, you will protect your health by reducing the likelihood of developing pancreatic cancer.  Remember to try and eat no more than two portions of red/processed meat this week.  A link to today’s food diary will be sent to you this evening so that you can record your food intake for today.  EVENING:  If you eat only a small amount of red and processed meat, you will protect your health by reducing the likelihood of developing pancreatic cancer.  Remember to try and eat no more than two portions of red/processed meat this week.  AFTER DINNER, please remember to record all of the food you eat today using today’s food diary.  Click “Go to Diary” to go to today’s food diary. Please make sure you press the red arrow at the end of the diary to save your responses. | MORNING:  If you eat only a small amount of red and processed meat, you will protect the environment by reducing air pollution.  Remember to try and eat no more than two portions of red/processed meat this week.  A link to today’s food diary will be sent to you this evening so that you can record your food intake for today.  EVENING:  If you eat only a small amount of red and processed meat, you will protect the environment by reducing air pollution.  Remember to try and eat no more than two portions of red/processed meat this week.  AFTER DINNER, please remember to record all of the food you eat today using today’s food diary.  Click “Go to Diary” to go to today’s food diary. Please make sure you press the red arrow at the end of the diary to save your responses. | MORNING:  If you eat only a small amount of red and processed meat, you will protect your health by reducing the likelihood of developing pancreatic cancer and you will protect the environment by reducing air pollution.  Remember to try and eat no more than two portions of red/processed meat this week.  A link to today’s food diary will be sent to you this evening so that you can record your food intake for today.  EVENING:  If you eat only a small amount of red and processed meat, you will protect your health by reducing the likelihood of developing pancreatic cancer and you will protect the environment by reducing air pollution.  Remember to try and eat no more than two portions of red/processed meat this week.  AFTER DINNER, please remember to record all of the food you eat today using today’s food diary.  Click “Go to Diary” to go to today’s food diary. Please make sure you press the red arrow at the end of the diary to save your responses. |
| MORNING:  A link to today’s food diary will be sent to you this evening so that you can record your food intake for today.  That is all for now. I will write to you tonight with further instructions.  EVENING:  AFTER DINNER, please remember to record all of the food you eat today using today’s food diary.  Click “Go to Diary” to go to today’s food diary. Please make sure you press the red arrow at the end of the diary to save your responses. | MORNING:  If you eat only a small amount of red and processed meat, you will protect your health by reducing the likelihood of developing stomach cancer.  Remember to try and eat no more than two portions of red/processed meat this week.  A link to today’s food diary will be sent to you this evening so that you can record your food intake for today.  EVENING:  If you eat only a small amount of red and processed meat, you will protect your health by reducing the likelihood of developing stomach cancer.  Remember to try and eat no more than two portions of red/processed meat this week.  AFTER DINNER, please remember to record all of the food you eat today using today’s food diary.  Click “Go to Diary” to go to today’s food diary. Please make sure you press the red arrow at the end of the diary to save your responses. | MORNING:  If you eat only a small amount of red and processed meat, you will protect the environment by reducing desertification.  Remember to try and eat no more than two portions of red/processed meat this week.  A link to today’s food diary will be sent to you this evening so that you can record your food intake for today.  EVENING:  If you eat only a small amount of red and processed meat, you will protect the environment by reducing desertification.  Remember to try and eat no more than two portions of red/processed meat this week.  AFTER DINNER, please remember to record all of the food you eat today using today’s food diary.  Click “Go to Diary” to go to today’s food diary. Please make sure you press the red arrow at the end of the diary to save your responses. | MORNING:  If you eat only a small amount of red and processed meat, you will protect the environment by reducing desertification and you will protect your health by reducing the likelihood of developing stomach cancer.  Remember to try and eat no more than two portions of red/processed meat this week.  A link to today’s food diary will be sent to you this evening so that you can record your food intake for today.  EVENING:  If you eat only a small amount of red and processed meat, you will protect the environment by reducing desertification and you will protect your health by reducing the likelihood of developing stomach cancer.  Remember to try and eat no more than two portions of red/processed meat this week.  AFTER DINNER, please remember to record all of the food you eat today using today’s food diary.  Click “Go to Diary” to go to today’s food diary. Please make sure you press the red arrow at the end of the diary to save your responses. |
| MORNING:  A link to today’s food diary will be sent to you this evening so that you can record your food intake for today.  That is all for now. I will write to you tonight with further instructions.  EVENING:  AFTER DINNER, please remember to record all of the food you eat today using today’s food diary.  Click “Go to Diary” to go to today’s food diary. Please make sure you press the red arrow at the end of the diary to save your responses. | MORNING:  If you eat only a small amount of red and processed meat, you will protect your health by reducing the likelihood of having a stroke.  Remember to try and eat no more than two portions of red/processed meat this week.  A link to today’s food diary will be sent to you this evening so that you can record your food intake for today.  EVENING:  If you eat only a small amount of red and processed meat, you will protect your health by reducing the likelihood of having a stroke.  Remember to try and eat no more than two portions of red/processed meat this week.  AFTER DINNER, please remember to record all of the food you eat today using today’s food diary.  Click “Go to Diary” to go to today’s food diary. Please make sure you press the red arrow at the end of the diary to save your responses. | MORNING:  If you eat only a small amount of red and processed meat, you will protect the environment by reducing land degradation.  Remember to try and eat no more than two portions of red/processed meat this week.  A link to today’s food diary will be sent to you this evening so that you can record your food intake for today.  EVENING:  If you eat only a small amount of red and processed meat, you will protect the environment by reducing land degradation.  Remember to try and eat no more than two portions of red/processed meat this week.  AFTER DINNER, please remember to record all of the food you eat today using today’s food diary.  Click “Go to Diary” to go to today’s food diary. Please make sure you press the red arrow at the end of the diary to save your responses. | MORNING:  If you eat only a small amount of red and processed meat you will protect your health by reducing the likelihood of having a stroke and you will protect the environment by reducing land degradation.  Remember to try and eat no more than two portions of red/processed meat this week.  A link to today’s food diary will be sent to you this evening so that you can record your food intake for today.  EVENING:  If you eat only a small amount of red and processed meat you will protect your health by reducing the likelihood of having a stroke and you will protect the environment by reducing land degradation.  Remember to try and eat no more than two portions of red/processed meat this week.  AFTER DINNER, please remember to record all of the food you eat today using today’s food diary.  Click “Go to Diary” to go to today’s food diary. Please make sure you press the red arrow at the end of the diary to save your responses. |
| MORNING:  A link to today’s food diary will be sent to you this evening so that you can record your food intake for today.  That is all for now. I will write to you tonight with further instructions.  EVENING:  AFTER DINNER, please remember to record all of the food you eat today using today’s food diary.  Click “Go to Diary” to go to today’s food diary. Please make sure you press the red arrow at the end of the diary to save your responses. | MORNING:  If you eat only a small amount of red and processed meat, you will protect your health by reducing the likelihood of developing liver disease.  Remember to try and eat no more than two portions of red/processed meat this week.  A link to today’s food diary will be sent to you this evening so that you can record your food intake for today.  EVENING:  If you eat only a small amount of red and processed meat, you will protect your health by reducing the likelihood of developing liver disease.  Remember to try and eat no more than two portions of red/processed meat this week.  AFTER DINNER, please remember to record all of the food you eat today using today’s food diary.  Click “Go to Diary” to go to today’s food diary. Please make sure you press the red arrow at the end of the diary to save your responses. | MORNING:  If you eat only a small amount of red and processed meat, you will protect the environment by reducing climate change.  Remember to try and eat no more than two portions of red/processed meat this week.  A link to today’s food diary will be sent to you this evening so that you can record your food intake for today.  EVENING:  If you eat only a small amount of red and processed meat, you will protect the environment by reducing climate change.  Remember to try and eat no more than two portions of red/processed meat this week.  AFTER DINNER, please remember to record all of the food you eat today using today’s food diary.  Click “Go to Diary” to go to today’s food diary. Please make sure you press the red arrow at the end of the diary to save your responses. | MORNING:  If you eat only a small amount of red and processed meat, you will protect the environment by reducing climate change and you will protect your health by reducing the likelihood of developing liver disease.  Remember to try and eat no more than two portions of red/processed meat this week.  A link to today’s food diary will be sent to you this evening so that you can record your food intake for today.  EVENING:  If you eat only a small amount of red and processed meat, you will protect the environment by reducing climate change and you will protect your health by reducing the likelihood of developing liver disease.  Remember to try and eat no more than two portions of red/processed meat this week.  AFTER DINNER, please remember to record all of the food you eat today using today’s food diary.  Click “Go to Diary” to go to today’s food diary. Please make sure you press the red arrow at the end of the diary to save your responses. |
| MORNING:  A link to today’s food diary will be sent to you this evening so that you can record your food intake for today.  That is all for now. I will write to you tonight with further instructions.  EVENING:  AFTER DINNER, please remember to record all of the food you eat today using today’s food diary.  Click “Go to Diary” to go to today’s food diary. Please make sure you press the red arrow at the end of the diary to save your responses. | MORNING:  If you eat only a small amount of red and processed meat, you will protect your health by reducing the likelihood of developing respiratory disease.  Remember to try and eat no more than two portions of red/processed meat this week.  A link to today’s food diary will be sent to you this evening so that you can record your food intake for today.  EVENING:  If you eat only a small amount of red and processed meat, you will protect your health by reducing the likelihood of developing respiratory disease.  Remember to try and eat no more than two portions of red/processed meat this week.  AFTER DINNER, please remember to record all of the food you eat today using today’s food diary.  Click “Go to Diary” to go to today’s food diary. Please make sure you press the red arrow at the end of the diary to save your responses. | MORNING:  If you eat only a small amount of red and processed meat, you will protect the environment by reducing soil acidification.  Remember to try and eat no more than two portions of red/processed meat this week.  A link to today’s food diary will be sent to you this evening so that you can record your food intake for today.  EVENING:  If you eat only a small amount of red and processed meat, you will protect the environment by reducing soil acidification.  Remember to try and eat no more than two portions of red/processed meat this week.  AFTER DINNER, please remember to record all of the food you eat today using today’s food diary.  Click “Go to Diary” to go to today’s food diary. Please make sure you press the red arrow at the end of the diary to save your responses. | MORNING:  If you eat only a small amount of red and processed meat, you will protect your health by reducing the likelihood of developing respiratory disease and you will protect the environment by reducing soil acidification.  Remember to try and eat no more than two portions of red/processed meat this week.  A link to today’s food diary will be sent to you this evening so that you can record your food intake for today.  EVENING:  If you eat only a small amount of red and processed meat, you will protect your health by reducing the likelihood of developing respiratory disease and you will protect the environment by reducing soil acidification.  Remember to try and eat no more than two portions of red/processed meat this week.  AFTER DINNER, please remember to record all of the food you eat today using today’s food diary.  Click “Go to Diary” to go to today’s food diary. Please make sure you press the red arrow at the end of the diary to save your responses. |
| MORNING:  A link to today’s food diary will be sent to you this evening so that you can record your food intake for today.  That is all for now. I will write to you tonight with further instructions.  EVENING:  AFTER DINNER, please remember to record all of the food you eat today using today’s food diary.  Click “Go to Diary” to go to today’s food diary. Please make sure you press the red arrow at the end of the diary to save your responses. | MORNING:  If you eat only a small amount of red and processed meat, you will protect your health by reducing the likelihood of developing kidney disease.  Remember to try and eat no more than two portions of red/processed meat this week.  A link to today’s food diary will be sent to you this evening so that you can record your food intake for today.  EVENING:  If you eat only a small amount of red and processed meat, you will protect your health by reducing the likelihood of developing kidney disease.  Remember to try and eat no more than two portions of red/processed meat this week.  AFTER DINNER, please remember to record all of the food you eat today using today’s food diary.  Click “Go to Diary” to go to today’s food diary. Please make sure you press the red arrow at the end of the diary to save your responses. | MORNING:  If you eat only a small amount of red and processed meat, you will protect the environment by reducing harmful changes to soil and land.  Remember to try and eat no more than two portions of red/processed meat this week.  A link to today’s food diary will be sent to you this evening so that you can record your food intake for today.  EVENING:  If you eat only a small amount of red and processed meat, you will protect the environment by reducing harmful changes to soil and land.    Remember to try and eat no more than two portions of red/processed meat this week.  AFTER DINNER, please remember to record all of the food you eat today using today’s food diary.  Click “Go to Diary” to go to today’s food diary. Please make sure you press the red arrow at the end of the diary to save your responses. | MORNING:  If you eat only a small amount of red and processed meat, you will protect the environment by reducing harmful changes to soil and land and you will protect your health by reducing the likelihood of developing kidney disease.  Remember to try and eat no more than two portions of red/processed meat this week.  A link to today’s food diary will be sent to you this evening so that you can record your food intake for today.  EVENING:  If you eat only a small amount of red and processed meat, you will protect the environment by reducing harmful changes to soil and land and you will protect your health by reducing the likelihood of developing kidney disease.  Remember to try and eat no more than two portions of red/processed meat this week.  AFTER DINNER, please remember to record all of the food you eat today using today’s food diary.  Click “Go to Diary” to go to today’s food diary. Please make sure you press the red arrow at the end of the diary to save your responses. |
| MORNING:  A link to today’s food diary will be sent to you this evening so that you can record your food intake for today.  That is all for now. I will write to you tonight with further instructions.  EVENING:  AFTER DINNER, please remember to record all of the food you eat today using today’s food diary.  Click “Go to Diary” to go to today’s food diary. Please make sure you press the red arrow at the end of the diary to save your responses. | MORNING:  If you eat only a small amount of red and processed meat, you will protect your health by reducing the likelihood of developing infections.  Remember to try and eat no more than two portions of red/processed meat this week.  A link to today’s food diary will be sent to you this evening so that you can record your food intake for today.  EVENING:  If you eat only a small amount of red and processed meat, you will protect your health by reducing the likelihood of developing infections.  Remember to try and eat no more than two portions of red/processed meat this week.  AFTER DINNER, please remember to record all of the food you eat today using today’s food diary.  Click “Go to Diary” to go to today’s food diary. Please make sure you press the red arrow at the end of the diary to save your responses. | MORNING:  If you eat only a small amount of red and processed meat, you will protect the environment by reducing the presence of microorganisms and dust in the air.  Remember to try and eat no more than two portions of red/processed meat this week.  A link to today’s food diary will be sent to you this evening so that you can record your food intake for today.  EVENING:  If you eat only a small amount of red and processed meat, you will protect the environment by reducing the presence of microorganisms and dust in the air.  Remember to try and eat no more than two portions of red/processed meat this week.  AFTER DINNER, please remember to record all of the food you eat today using today’s food diary.  Click “Go to Diary” to go to today’s food diary. Please make sure you press the red arrow at the end of the diary to save your responses. | MORNING:  If you eat only a small amount of red and processed meat, you will protect your health by reducing the likelihood of developing infections and you will protect the environment by reducing the presence of microorganisms and dust in the air.  Remember to try and eat no more than two portions of red/processed meat this week.  A link to today’s food diary will be sent to you this evening so that you can record your food intake for today.  EVENING:  If you eat only a small amount of red and processed meat, you will protect your health by reducing the likelihood of developing infections and you will protect the environment by reducing the presence of microorganisms and dust in the air.  Remember to try and eat no more than two portions of red/processed meat this week.  AFTER DINNER, please remember to record all of the food you eat today using today’s food diary.  Click “Go to Diary” to go to today’s food diary. Please make sure you press the red arrow at the end of the diary to save your responses. |

**Supplementary Tables**

Table S1

*Regression parameters of change in red and processed meat consumption (T2 – T1) as a predictor of participants’ willingness to engage in pro-environmental behaviours at T2 with dummy coded conditions*

|  | ***B*** | ***SE (B)*** | ***β*** | ***t*** | ***p*** |
| --- | --- | --- | --- | --- | --- |
| Have shorter showers or infrequent baths | | | | | |
| Change in red and processed meat consumption | 0.01 | 0.03 | 0.01 | 0.20 | .839 |
| Health vs. control condition | 0.21 | 0.42 | 0.04 | 0.50 | .615 |
| Environment vs. control condition | -0.32 | 0.40 | -0.07 | -0.79 | .429 |
| Combined vs. control condition | -0.01 | 0.40 | -0.00 | -0.02 | .981 |
| Buy an eco-friendly product | | | | | |
| Change in red and processed meat consumption | -0.01 | 0.03 | -0.02 | -0.26 | .794 |
| Health vs. control condition | 0.08 | 0.37 | 0.02 | 0.21 | .836 |
| Environment vs. control condition | -0.25 | 0.35 | -0.06 | -0.71 | .478 |
| Combined vs. control condition | 0.01 | 0.35 | 0.00 | 0.01 | .989 |
| Buy a product with less packaging | | | | | |
| Change in red and processed meat consumption | 0.00 | 0.03 | 0.01 | 0.15 | .881 |
| Health vs. control condition | -0.12 | 0.37 | -0.03 | -0.34 | .735 |
| Environment vs. control condition | -0.72 | 0.35 | -0.17 | -2.04 | .042 |
| Combined vs. control condition | -0.40 | 0.35 | -0.10 | -1.15 | .253 |
| Buy organic food produce | | | | | |
| Change in red and processed meat consumption | -0.02 | 0.03 | -0.04 | -0.65 | .516 |
| Health vs. control condition | -0.62 | 0.39 | -0.13 | -1.59 | .114 |
| Environment vs. control condition | -0.44 | 0.37 | -0.10 | -1.19 | .236 |
| Combined vs. control condition | 0.02 | 0.37 | 0.00 | 0.05 | .963 |
| Buy local rather than imported food produce | | | | | |
| Change in red and processed meat consumption | -0.01 | 0.03 | -0.03 | -0.41 | .686 |
| Health vs. control condition | 0.21 | 0.39 | 0.05 | 0.56 | .579 |
| Environment vs. control condition | -0.08 | 0.37 | -0.02 | -0.22 | .826 |
| Combined vs. control condition | 0.06 | 0.37 | 0.01 | 0.16 | .873 |
| Eat seasonal fruit and vegetables | | | | | |
| Change in red and processed meat consumption | -0.01 | 0.03 | -0.03 | -0.48 | .633 |
| Health vs. control condition | 0.10 | 0.34 | 0.02 | 0.29 | .769 |
| Environment vs. control condition | -0.22 | 0.33 | -0.06 | -0.67 | .506 |
| Combined vs. control condition | 0.06 | 0.33 | 0.02 | 0.19 | .852 |
| Reduce my consumption of meat and dairy products | | | | | |
| Change in red and processed meat consumption | -0.07 | 0.03 | -0.16 | -2.38 | .018 |
| Health vs. control condition | 0.34 | 0.37 | 0.08 | 0.93 | .356 |
| Environment vs. control condition | 0.49 | 0.35 | 0.11 | 1.38 | .169 |
| Combined vs. control condition | 0.24 | 0.35 | 0.06 | 0.67 | .501 |
| Use public transport instead of driving my car | | | | | |
| Change in red and processed meat consumption | -0.05 | 0.03 | -0.10 | -1.52 | .131 |
| Health vs. control condition | -0.45 | 0.41 | -0.09 | -1.10 | .272 |
| Environment vs. control condition | -0.74 | 0.39 | -0.16 | -1.89 | .060 |
| Combined vs. control condition | -0.08 | 0.39 | -0.02 | -0.20 | .840 |
| Volunteer for environmental group | | | | | |
| Change in red and processed meat consumption | 0.03 | 0.02 | 0.08 | 1.15 | .251 |
| Health vs. control condition | 0.29 | 0.28 | 0.09 | 1.02 | .310 |
| Environment vs. control condition | 0.19 | 0.27 | 0.06 | 0.71 | .479 |
| Combined vs. control condition | 0.26 | 0.27 | 0.08 | 0.94 | .346 |
| Donate to an environmental group | | | | | |
| Change in red and processed meat consumption | 0.03 | 0.02 | 0.11 | 1.62 | .107 |
| Health vs. control condition | 0.42 | 0.26 | 0.13 | 1.61 | .109 |
| Environment vs. control condition | 0.17 | 0.25 | 0.06 | 0.70 | .484 |
| Combined vs. control condition | 0.26 | 0.25 | 0.09 | 1.03 | .305 |

Table S2

*Regression parameters of change in red and processed meat consumption (T3 – T1) as a predictor of participants’ willingness to engage in pro-environmental behaviours at T3 with dummy coded conditions*

|  | ***B*** | ***SE (B)*** | ***β*** | ***t*** | ***p*** |
| --- | --- | --- | --- | --- | --- |
| Have shorter showers or infrequent baths | | | | | |
| Change in red and processed meat consumption | -0.06 | 0.04 | -0.12 | -1.77 | .079 |
| Health vs. control condition | 0.26 | 0.40 | 0.05 | 0.64 | .520 |
| Environment vs. control condition | -0.42 | 0.39 | -0.09 | -1.09 | .277 |
| Combined vs. control condition | 0.25 | 0.40 | 0.05 | 0.63 | .530 |
| Purchase an eco-friendly product | | | | | |
| Change in red and processed meat consumption | -0.07 | 0.03 | -0.15 | -2.29 | .023 |
| Health vs. control condition | -0.12 | 0.34 | -0.03 | -0.36 | .720 |
| Environment vs. control condition | -0.39 | 0.32 | -0.10 | -1.22 | .225 |
| Combined vs. control condition | 0.07 | 0.33 | 0.02 | 0.21 | .832 |
| Buy a product with less packaging | | | | | |
| Change in red and processed meat consumption | -0.08 | 0.03 | -0.16 | -2.44 | .016 |
| Health vs. control condition | 0.02 | 0.37 | 0.01 | 0.06 | .955 |
| Environment vs. control condition | -0.32 | 0.35 | -0.08 | -0.91 | .365 |
| Combined vs. control condition | -0.12 | 0.36 | -0.03 | -0.33 | .742 |
| Buy organic food produce | | | | | |
| Change in red and processed meat consumption | -0.04 | 0.03 | -0.08 | -1.25 | .214 |
| Health vs. control condition | -0.21 | 0.38 | -0.05 | -0.54 | .587 |
| Environment vs. control condition | -0.35 | 0.36 | -0.08 | -0.97 | .334 |
| Combined vs. control condition | 0.28 | 0.37 | 0.07 | 0.76 | .449 |
| Buy local rather than imported food produce | | | | | |
| Change in red and processed meat consumption | -0.08 | 0.03 | -0.18 | -2.66 | .008 |
| Health vs. control condition | 0.15 | 0.35 | 0.03 | 0.41 | .682 |
| Environment vs. control condition | -0.15 | 0.34 | -0.04 | -0.45 | .655 |
| Combined vs. control condition | 0.12 | 0.35 | 0.03 | 0.34 | .733 |
| Eat seasonal fruit and vegetables | | | | | |
| Change in red and processed meat consumption | -0.10 | 0.03 | -0.21 | -3.21 | .001 |
| Health vs. control condition | -0.40 | 0.36 | -0.09 | -1.12 | .264 |
| Environment vs. control condition | -0.30 | 0.34 | -0.07 | -0.87 | .383 |
| Combined vs. control condition | 0.05 | 0.35 | 0.01 | 0.15 | .880 |
| Reduce my consumption of meat and dairy products | | | | | |
| Change in red and processed meat consumption | -0.08 | 0.03 | -0.15 | -2.39 | .018^*^ |
| Health vs. control condition | 0.26 | 0.37 | 0.06 | 0.69 | .491 |
| Environment vs. control condition | 1.00 | 0.36 | 0.23 | 2.80 | .006^*^ |
| Combined vs. control condition | 1.07 | 0.37 | 0.24 | 2.91 | .004^*^ |
| Environment vs. health condition | 0.74 | 0.34 | 0.17 | 2.15 | .032^*^ |
| Combined vs. health condition | 0.81 | 0.35 | 0.18 | 2.31 | .022^*^ |
| Environment vs. combined | 0.07 | 0.33 | 0.02 | 0.20 | .841 |
| Use public transport instead of driving my car | | | | | |
| Change in red and processed meat consumption | -0.07 | 0.04 | -0.12 | -1.87 | .063 |
| Health vs. control condition | 0.11 | 0.43 | 0.02 | 0.26 | .795 |
| Environment vs. control condition | -0.30 | 0.41 | -0.06 | -0.74 | .460 |
| Combined vs. control condition | -0.24 | 0.42 | -0.05 | -0.58 | .563 |
| Volunteer for environmental group | | | | | |
| Change in red and processed meat consumption | 0.00 | 0.02 | 0.01 | 0.21 | .831 |
| Health vs. control condition | 0.09 | 0.24 | 0.03 | 0.38 | .707 |
| Environment vs. control condition | 0.14 | 0.23 | 0.05 | 0.60 | .552 |
| Combined vs. control condition | 0.23 | 0.23 | 0.09 | 0.98 | .329 |
| Donate to an environmental group | | | | | |
| Change in red and processed meat consumption | 0.01 | 0.02 | 0.04 | 0.63 | .529 |
| Health vs. control condition | 0.08 | 0.21 | 0.03 | 0.37 | .708 |
| Environment vs. control condition | 0.09 | 0.20 | 0.04 | 0.46 | .647 |
| Combined vs. control condition | 0.37 | 0.21 | 0.15 | 1.76 | .079 |

Note. ^*^ p significant after Holm-Bonferroni correction.

Table S3

*Multiple regression parameters of change in red and processed meat consumption (T2 – T1) as a predictor of change in pro-environmental identity* (*T2 – T1) with dummy coded conditions*

|  |  | ***B*** | ***SE (B)*** | ***β*** | ***t*** | ***p*** |
| --- | --- | --- | --- | --- | --- | --- |
| Model 1 | Baseline pro-environmental identity | -0.29 | 0.04 | -0.41 | -6.97 | .000^*^ |
| Model 2 | Baseline pro-environmental identity | -0.30 | 0.04 | -0.41 | -7.00 | .000^*^ |
|  | Change in red and processed meat consumption | -0.01 | 0.01 | -0.04 | -0.67 | .502 |
|  | Health vs. control condition | -0.10 | 0.13 | -0.06 | -0.77 | .442 |
|  | Environment vs. control condition | -0.12 | 0.13 | -0.07 | -0.93 | .353 |
|  | Combined vs. control condition | -0.14 | 0.13 | -0.08 | -1.07 | .284 |

Note. ^*^ p significant after Holm-Bonferroni correction.

Table S4

*Multiple regression parameters of change in red and processed meat consumption (T3 – T1) as a predictor of change in pro-environmental identity (T3 – T1) with dummy coded conditions*

|  |  | ***B*** | ***SE (B)*** | ***β*** | ***t*** | ***p*** |
| --- | --- | --- | --- | --- | --- | --- |
| Model 1 | Baseline pro-environmental identity | -0.39 | 0.05 | -0.46 | -8.01 | .000^*^ |
| Model 2 | Baseline pro-environmental identity | -0.40 | 0.05 | -0.47 | -8.16 | .000^*^ |
|  | Change in red and processed meat consumption | 0.01 | 0.01 | 0.02 | 0.41 | .682 |
|  | Health vs. control condition | -0.07 | 0.15 | -0.03 | -0.45 | .653 |
|  | Environment vs. control condition | -0.24 | 0.14 | -0.13 | -1.72 | .087 |
|  | Combined vs. control condition | -0.03 | 0.15 | -0.01 | -0.19 | .846 |

Note. ^*^ p significant after Holm-Bonferroni correction.

Table S5

*Regression parameters of change in pro-environmental identity (T2 – T1) as a predictor of participants’ willingness to engage in pro-environmental behaviours at T2 with dummy coded conditions*

|  | ***B*** | ***SE (B)*** | ***β*** | ***t*** | ***p*** |
| --- | --- | --- | --- | --- | --- |
| Have shorter showers or infrequent baths | | | | | |
| Change in pro-environmental identity | 0.27 | 0.18 | 0.10 | 1.50 | .135 |
| Health vs. control | 0.19 | 0.40 | 0.04 | 0.48 | .628 |
| Environment vs. control | -0.33 | 0.39 | -0.07 | -0.86 | .390 |
| Combined vs. control | -0.01 | 0.38 | -0.00 | -0.04 | .970 |
| Purchase an eco-friendly product | | | | | |
| Change in pro-environmental identity | 0.19 | 0.16 | 0.08 | 1.17 | .242 |
| Health vs. control | 0.11 | 0.35 | 0.02 | 0.31 | .760 |
| Environment vs. control | -0.23 | 0.34 | -0.05 | -0.67 | .503 |
| Combined vs. control | 0.04 | 0.34 | 0.01 | 0.12 | .905 |
| Buy a product with less packaging | | | | | |
| Change in pro-environmental identity | 0.08 | 0.16 | 0.03 | 0.51 | .609 |
| Health vs. control | -0.14 | 0.35 | -0.03 | -0.39 | .697 |
| Environment vs. control | -0.73 | 0.34 | -0.17 | -2.13 | .034 |
| Combined vs. control | -0.41 | 0.34 | -0.10 | -1.21 | .226 |
| Buy organic food produce | | | | | |
| Change in pro-environmental identity | -0.11 | 0.17 | -0.04 | -0.66 | .507 |
| Health vs. control | -0.55 | 0.37 | -0.12 | -1.47 | .143 |
| Environment vs. control | -0.39 | 0.36 | -0.09 | -1.07 | .285 |
| Combined vs. control | 0.07 | 0.36 | 0.02 | 0.21 | .837 |
| Buy local rather than imported food produce | | | | | |
| Change in pro-environmental identity | -0.02 | 0.17 | -0.01 | -0.12 | .908 |
| Health vs. control | 0.26 | 0.37 | 0.06 | 0.70 | .488 |
| Environment vs. control | -0.05 | 0.36 | -0.01 | -0.14 | .893 |
| Combined vs. control | 0.10 | 0.36 | 0.02 | 0.27 | .786 |
| Eat seasonal fruit and vegetables | | | | | |
| Change in pro-environmental identity | 0.11 | 0.15 | 0.05 | 0.70 | .482 |
| Health vs. control | 0.15 | 0.33 | 0.04 | 0.45 | .650 |
| Environment vs. control | -0.18 | 0.32 | -0.05 | -0.58 | .565 |
| Combined vs. control | 0.11 | 0.31 | 0.03 | 0.34 | .731 |
| Reduce my consumption of meat and dairy products | | | | | |
| Change in pro-environmental identity | 0.25 | 0.16 | 0.10 | 1.53 | .128 |
| Health vs. control | 0.59 | 0.36 | 0.13 | 1.67 | .097 |
| Environment vs. control | 0.67 | 0.35 | 0.15 | 0.19 | .054 |
| Combined vs. control | 0.47 | 0.34 | 0.11 | 1.38 | .169 |
| Use public transport instead of driving my car | | | | | |
| Change in pro-environmental identity | 0.21 | 0.18 | 0.07 | 1.15 | .253 |
| Health vs. control | -0.27 | 0.39 | -0.06 | -0.69 | .491 |
| Environment vs. control | -0.61 | 0.38 | -0.13 | -1.59 | .113 |
| Combined vs. control | 0.09 | 0.38 | 0.02 | 0.24 | .814 |
| Volunteer for environmental group | | | | | |
| Change in pro-environmental identity | 0.09 | 0.13 | 0.05 | 0.70 | .487 |
| Health vs. control | 0.20 | 0.27 | 0.06 | 0.73 | .465 |
| Environment vs. control | 0.12 | 0.27 | 0.04 | 0.47 | .640 |
| Combined vs. control | 0.18 | 0.26 | 0.06 | 0.68 | .495 |
| Donate to an environmental group | | | | | |
| Change in pro-environmental identity | 0.14 | 0.11 | 0.08 | 1.19 | .235 |
| Health vs. control | 0.30 | 0.25 | 0.10 | 1.21 | .226 |
| Environment vs. control | 0.09 | 0.24 | 0.03 | 0.36 | .723 |
| Combined vs. control | 0.16 | 0.24 | 0.05 | 0.66 | .511 |

Table S6

*Regression parameters of change in pro-environmental identity (T3 – T1) as a predictor of participants’ willingness to engage in pro-environmental behaviours at T3 with dummy coded conditions*

|  | ***B*** | ***SE (B)*** | ***β*** | ***t*** | ***p*** |
| --- | --- | --- | --- | --- | --- |
| Have shorter showers or infrequent baths | | | | | |
| Change in pro-environmental identity | 0.15 | 0.16 | 0.06 | 0.97 | .332 |
| Health vs. control | 0.35 | 0.40 | 0.07 | 0.87 | .385 |
| Environment vs. control | -0.33 | 0.39 | -0.07 | -0.85 | .397 |
| Combined vs. control | 0.37 | 0.39 | 0.08 | 0.95 | .343 |
| Purchase an eco-friendly product | | | | | |
| Change in pro-environmental identity | 0.01 | 0.13 | 0.00 | 0.05 | .964 |
| Health vs. control | -0.03 | 0.34 | -0.01 | -0.08 | .935 |
| Environment vs. control | -0.32 | 0.33 | -0.08 | -0.98 | .326 |
| Combined vs. control | 0.20 | 0.33 | 0.05 | 0.60 | .553 |
| Buy a product with less packaging | | | | | |
| Change in pro-environmental identity | 0.19 | 0.14 | 0.09 | 1.33 | .184 |
| Health vs. control | 0.13 | 0.37 | 0.03 | 0.37 | .715 |
| Environment vs. control | -0.20 | 0.35 | -0.05 | -0.57 | .566 |
| Combined vs. control | 0.04 | 0.36 | 0.01 | 0.10 | .918 |
| Buy organic food produce | | | | | |
| Change in pro-environmental identity | -0.23 | 0.15 | -0.10 | -1.55 | .122 |
| Health vs. control | -0.16 | 0.38 | -0.03 | -0.41 | .679 |
| Environment vs. control | -0.35 | 0.36 | -0.08 | -0.97 | .335 |
| Combined vs. control | 0.35 | 0.37 | 0.08 | 0.97 | .345 |
| Buy local rather than imported food produce | | | | | |
| Change in pro-environmental identity | 0.14 | 0.14 | 0.07 | 1.04 | .301 |
| Health vs. control | 0.26 | 0.36 | 0.06 | 0.74 | .461 |
| Environment vs. control | -0.04 | 0.34 | -0.01 | -0.12 | .907 |
| Combined vs. control | 0.28 | 0.35 | 0.07 | 0.81 | .422 |
| Eat seasonal fruit and vegetables | | | | | |
| Change in pro-environmental identity | -0.10 | 0.14 | -0.05 | -0.73 | .466 |
| Health vs. control | -0.26 | 0.36 | -0.06 | -0.73 | .466 |
| Environment vs. control | -0.21 | 0.35 | -0.05 | -0.61 | .543 |
| Combined vs. control | 0.23 | 0.35 | 0.06 | 0.66 | .508 |
| Reduce my consumption of meat and dairy products | | | | | |
| Change in pro-environmental identity | -0.02 | 0.15 | -0.01 | -0.13 | .898 |
| Health vs. control | 0.37 | 0.38 | 0.08 | 0.97 | .332 |
| Environment vs. control | 1.08 | 0.36 | 0.25 | 2.99 | .003 |
| Combined vs. control | 1.21 | 0.37 | 0.27 | 3.31 | .001 |
| Use public transport instead of driving my car | | | | | |
| Change in pro-environmental identity | -0.12 | 0.17 | -0.05 | -0.72 | .470 |
| Health vs. control | 0.21 | 0.43 | 0.04 | 0.48 | .633 |
| Environment vs. control | -0.25 | 0.41 | -0.05 | -0.61 | .541 |
| Combined vs. control | -0.12 | 0.42 | -0.02 | -0.29 | .774 |
| Volunteer for environmental group | | | | | |
| Change in pro-environmental identity | 0.07 | 0.09 | 0.05 | 0.77 | .442 |
| Health vs. control | 0.09 | 0.24 | 0.03 | 0.36 | .717 |
| Environment vs. control | 0.14 | 0.23 | 0.05 | 0.63 | .527 |
| Combined vs. control | 0.22 | 0.23 | 0.08 | 0.97 | .331 |
| Donate to an environmental group | | | | | |
| Change in pro-environmental identity | 0.06 | 0.08 | 0.05 | 0.68 | .498 |
| Health vs. control | 0.06 | 0.21 | 0.03 | 0.31 | .758 |
| Environment vs. control | 0.09 | 0.20 | 0.04 | 0.45 | .655 |
| Combined vs. control | 0.35 | 0.20 | 0.14 | 1.70 | .091 |
